# Deletion of a Single LeishIF4E-3 Allele by the CRISPR-Cas9 System Alters Cell Morphology and Infectivity of *Leishmania*

**DOI:** 10.1128/mSphere.00450-19

**Published:** 2019-09-04

**Authors:** Rohit Shrivastava, Nitin Tupperwar, Matan Drory-Retwitzer, Michal Shapira

**Affiliations:** a Department of Life Sciences, Ben-Gurion University of the Negev, Beer-Sheva, Israel; b Department of Computer Sciences, Ben-Gurion University of the Negev, Beer-Sheva, Israel; The Hebrew University of Jerusalem

**Keywords:** CRISPR, CRISPR-Cas9, LeishIF4E-3, *Leishmania*, translation

## Abstract

The genomes of *Leishmania* and trypanosomes encode six paralogs of the eIF4E cap-binding protein, known in other eukaryotes to anchor the translation initiation complex. In line with the heteroxenous nature of these parasites, the different LeishIF4E paralogs vary in their biophysical features and their biological behavior. We therefore hypothesize that each has a specialized function, not limited to protein synthesis. Of the six paralogs, LeishIF4E-3 has a weak cap-binding activity. It participates in the assembly of granules that store inactive transcripts and ribosomal proteins during nutritional stress that is experienced in the sand fly. We investigated the role of LeishIF4E-3 in Leishmania mexicana promastigotes using the CRISPR-Cas9 system. We deleted one of the two LeishIF4E-3 alleles, generating a heterologous deletion mutant with reduced LeishIF4E-3 expression. The mutant showed a decline in *de novo* protein synthesis and growth kinetics, altered morphology, and impaired infectivity. The mutant cells were rounded and failed to transform into the nectomonad-like form, in response to purine starvation. Furthermore, the infectivity of macrophage cells by the LeishIF4E-3(+/−) mutant was severely reduced. These phenotypic features were not observed in the addback cells, in which expression of LeishIF4E-3 was restored. The observed phenotypic changes correlated with the profile of transcripts associated with LeishIF4E-3. These were enriched for cytoskeleton- and flagellum-encoding genes, along with genes for RNA binding proteins. Our data illustrate the importance of LeishIF4E-3 in translation and in the parasite virulence.

**IMPORTANCE** *Leishmania* species are the causative agents of a spectrum of diseases. Available drug treatment is toxic and expensive, with drug resistance a growing concern. *Leishmania* parasites migrate between transmitting sand flies and mammalian hosts, experiencing unfavorable extreme conditions. The parasites therefore developed unique mechanisms for promoting a stage-specific program for gene expression, with translation playing a central role. There are six paralogs of the cap-binding protein eIF4E, which vary in their function, expression profiles, and assemblages. Using the CRISPR-Cas9 system for *Leishmania*, we deleted one of the two LeishIF4E-3 alleles. Expression of LeishIF4E-3 in the deletion mutant was low, leading to reduction in global translation and growth of the mutant cells. Cell morphology also changed, affecting flagellum growth, cell shape, and infectivity. The importance of this study is in highlighting that LeishIF4E-3 is essential for completion of the parasite life cycle. Our study gives new insight into how parasite virulence is determined.

## Abstract (precis)

*Leishmania* species are the causative agents of a spectrum of diseases. Available drug treatment is toxic and expensive, with drug resistance a growing concern. *Leishmania* parasites migrate between transmitting sand flies and mammalian hosts, experiencing unfavorable extreme conditions. The parasites therefore developed unique mechanisms for promoting a stage-specific program for gene expression, with translation playing a central role. There are six paralogs of the cap-binding protein eIF4E, which vary in their function, expression profiles, and assemblages. Using the CRISPR-Cas9 system for *Leishmania*, we deleted one of the two LeishIF4E-3 alleles. Expression of LeishIF4E-3 in the deletion mutant was low, leading to reduction in global translation and growth of the mutant cells. Cell morphology also changed, affecting flagellum growth, cell shape, and infectivity. The importance of this study is in highlighting that LeishIF4E-3 is essential for completion of the parasite life cycle. Our study gives new insight into how parasite virulence is determined.

## INTRODUCTION

*Leishmania* parasites cycle between sand fly vectors and mammalian hosts, transforming from flagellated promastigotes to nonmotile obligatory intracellular parasites (1). Prior to their transmission by the female sand fly, the parasites differentiate from nonvirulent procyclic promastigotes first to intermediate nectomonad forms, which are typically long and slender, highly motile, and equipped with a relatively long flagellum. The parasites further differentiate to the virulent metacyclic forms (1–3). Metacyclogenesis is promoted by a nutritional stress that is imposed on the parasites in the intestinal tract of their vector, due to the deficiency of purines (4). *Leishmania* parasites lack the *de novo* biosynthetic pathway for purines and are therefore fully dependent on their supply from external sources (5–8). Transition between the life forms is promoted by a differential program of gene expression, with translation playing a key role (9).

Cap-dependent translation is the default mechanism for translation initiation. In eukaryotes, initiation proceeds following assembly of a cap-binding complex, eIF4F, on the cap structure at the 5′ end of the mRNA, anchoring the translation initiation complex. The eIF4F complex consists of a cap-binding protein (eIF4E), a DEAD box RNA helicase (eIF4A), and a scaffold protein (eIF4G) that holds together the complex (10). However, cap-dependent translation is often inhibited in a global manner, allowing translation of a limited repertoire of transcripts to initiate through cap-independent pathways (11, 12). *Leishmania* and other trypanosomatids encode six eIF4E paralogs and five eIF4G candidates. These show a high degree of sequence divergence, compared to their eukaryote counterparts and among themselves (13–15). Such variability could suggest that the different factors are involved in specific processes. Deciphering their role should therefore contribute to our understanding of the mechanisms by which the parasites adapt to the changing environmental conditions that occur throughout the life cycle. We previously reported that exposure of *Leishmania* parasites to different nutritional deprivations results in decreased translation and in formation of granules that store inactive ribosomal proteins and mRNAs, along with RNA helicases and RNA binding proteins. LeishIF4E-3 is one of the six *Leishmania* paralogs of eIF4Es concentrated in these granules (16). We show here that LeishIF4E-3 is an intriguing cap-binding protein, since despite its weak cap-binding activity (13), it is required for global translation under normal conditions.

In addition to the plethora of biochemical methodologies used to decipher the function of specific proteins, gene silencing or gene deletion is a fundamental tool. Gene silencing by RNA interference (RNAi) has been in practice for Trypanosoma brucei for over a decade (17), but a parallel system for *Leishmania* was not available (18, 19). The recent advancement of the CRISPR-Cas9 system has revolutionized the ability to examine the functions of target genes in general, and a CRISPR-Cas9 system was developed for *Leishmania*.

We deleted a single LeishIF4E-3 allele and show here that such deletion reduced the steady-state expression of the protein. This also affected cell morphology and infectivity, an effect that was assigned to the reduced expression of LeishIF4E-3, since the phenotypic changes were not observed in the addback strain.

## RESULTS

### Deletion of a single LeishIF4E-3 allele by CRISPR-Cas9 leads to reduced expression of the target protein.

To investigate the role of LeishIF4E-3 during the parasite life cycle and to examine whether it is essential for survival, we attempted to create a LeishIF4E-3 null mutant using two single guide RNAs (sgRNAs) that generate DNA breaks on either sides of the target gene, thereby replacing it with two repair cassettes containing resistance genes for G418 and blasticidin. However, only G418-resistant cells were obtained. These were further analyzed by PCR, to validate the deletion of LeishIF4E-3 and its replacement by the drug resistance marker.

**FIG 1:**
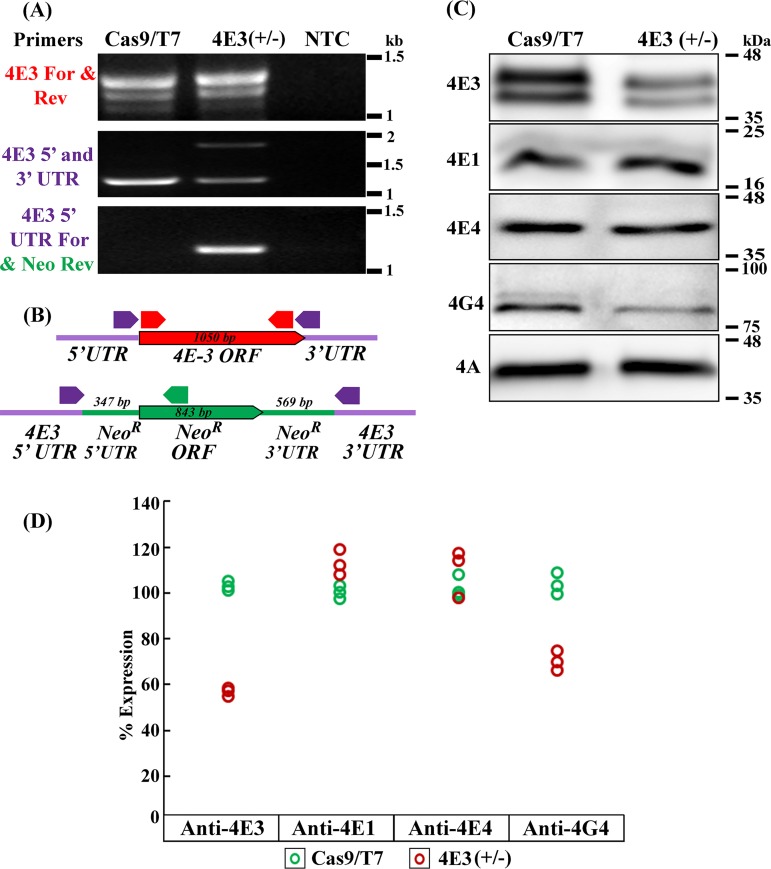
Generation of a LeishIF4E-3(+/−) deletion mutant by the CRISPR-Cas9 system. (A) A diagnostic PCR was carried out on the genomic DNA of L. mexicana, the transgenic parasite expressing Cas9/T7, and the LeishIF4E-3(+/−) deletion mutant using primers from the LeishIF4E-3 ORF, the 3′ and 5′ UTRs of LeishIF4E-3, and the 3′ UTR of LeishIF4E-3 with the reverse primer from the Neo^r^ ORF. A nontemplate control (NTC) was set up in parallel to exclude false positives. (B) Scheme showing the LeishIF4E-3 locus and the PCR primers (arrows) used to test the presence or absence of the LeishIF4E-3 coding sequence and the integration of the drug resistance cassette. Primers derived from the LeishIF4E-3 ORF are shown in red. Primers derived from the LeishIF4E-3 endogenous UTRs are shown in purple, and the primer derived from the Neo gene is shown in green. (C) A Western blot assay of LeishIF4E-3(+/−) mutants and control Cas9/T7 cell extracts separated by 12% SDS-PAGE and reacted with antibodies against LeishIF4E-3, LeishIF4E-1, LeishIF4E-4, LeishIF4G-4, and LeishIF4A-1 (the last served as a loading control). (D) Densitometric quantification of the bands corresponding to LeishIF4E-3, LeishIF4E-1, LeishIF4E-4, and LeishIF4G-4 in the deletion mutant LeishIF4E-3(+/−) (red) and the transgenic parasites expressing Cas9/T7 (green), shown in panel C. Expression of LeishIF4E-3, LeishIF4G-4, and LeishIF4A-1 was measured by Multi Gauge version 2.0 software. Expression of LeishIF4E-3, LeishIF4E-1, LeishIF4E-4, and LeishIF4G-4 was normalized to LeishIF4A-1 and is presented as a dot plot.

Diagnostic PCR using primers derived from the LeishIF4E-3 open reading frame (ORF) confirmed the presence of an intact LeishIF4E-3 gene copy (Fig. 1A, top panel). Another PCR using LeishIF4E-3 primers derived from the 5′ and 3′ untranslated regions (UTRs) generated two products (Fig. 1A, middle panel). One corresponded in size to the endogenous LeishIF4E-3 gene (∼1,050 bp), and a second product was larger (∼1,880 bp) and was presumably derived from the replacement G418 resistance gene, which was integrated between the 5′ and 3′ LeishIF4E-3 UTRs. Integration of the G418 replacement cassette in the proper target site in the genome while replacing the LeishIF4E-3 open reading frame was verified by a PCR with primers derived from the LeishIF4E-3 5′ UTR (forward) and from the open reading frame of the G418 resistance gene (reverse) (Fig. 1A, bottom panel). In conclusion, the PCR diagnosis confirmed the deletion of only a single allele of LeishIF4E-3. In an attempt to generate a null mutant of LeishIF4E-3, we repeated the cotransfection with two sgRNA templates and a repair cassette that contained the drug resistance gene for blasticidin. Successfully transfected cells did not survive the selection with 20 μg/ml blasticidin hydrochloride.

The effect of the LeishIF4E-3 heterologous deletion on expression of the target protein was evaluated by Western analysis of cell extracts derived from the LeishIF4E-3(+/−) mutant and from control cells transfected with the Cas9/T7 plasmid. The blots were reacted with antibodies against LeishIF4E-3 and LeishIF4A-1, the latter serving as a loading control. Figure 1B shows that although expression of LeishIF4E-3 was detected in the deletion mutant, it was reduced by 44% compared to the control cells that expressed only the Cas9/T7 genes and carried both alleles.

Since deletion of a protein can often lead to compensatory upregulation of other proteins that share the same complex, the blots were also reacted with antibodies against LeishIF4G-4, known as the specific binding partner of LeishIF4E-3 (20). As expected, the reduction in LeishIF4E-3 expression in the heterologous deletion mutant was accompanied by a parallel decrease in expression of LeishIF4G-4, suggesting that their expression is coordinated. However, other translation initiation factors such as LeishIF4E-1 and LeishIF4E-4 did not show a significant change in their steady-state expression (Fig. 1C and D). Western analysis of protein lysates derived from wild type and Cas9/T7 overexpression cells demonstrated no significant change in the steady-state expression of the tested proteins (see Fig. S1A in the supplemental material).

10.1128/mSphere.00450-19.2 FIG S1 Western analysis of steady-state expression of LeishIF4E-3. (A) (I) Cell extracts of wild-type L. mexicana and the transgenic parasite expressing Cas9/T7 were resolved by 12% SDS-PAGE and subjected to Western blot analysis using antibodies against LeishIF4E-3, LeishIF4G-4, and LeishIF4A-1. The last served as a loading control. (II) Densitometric analysis of the Western blot monitoring LeishIF4E-3 and LeishIF4G-4 expression from panel I. Antibody reactivity with each protein was fully quantified using the Multi Gauge software, version 2.0. The values were normalized to the protein loads and represented as dot plots. (B) Western analysis of LeishIF4E-3 expression following complementation in addback parasites. (I) Extracts of the mutant LeishIF4E-3(+/−), LeishIF4E-3 addback, wild-type L. mexicana, and transgenic parasite expressing Cas9/T7 were resolved by 12% SDS-PAGE and subjected to Western blot analysis using antibodies against LeishIF4E-3 and against SBP tag. Equal protein loads were verified by Ponceau staining of the blots. (II) Densitometric analysis was performed using Multi Gauge software, version 2.0. The values were normalized to the protein load and are shown as dots. Download FIG S1, PDF file, 0.3 MB. Copyright © 2019 Shrivastava et al. 2019 Shrivastava et al. This content is distributed under the terms of the Creative Commons Attribution 4.0 International license.

Expression of LeishIF4E-3 in the mutant cells was restored, to examine whether this reversed the overall effects observed in the LeishIF4E-3(+/−) deletion mutant. The cells were transfected with the pT-H-PuroLeishIF4E-3-SBP-H plasmid. The added-back LeishIF4E-3 gene was tagged with streptavidin binding protein (SBP), to distinguish it from the endogenous protein. Wild-type cells and Cas9/T7 expressers were included for control in the Western analysis. The restored expression of the added-back LeishIF4E-3-SBP was verified by Western analysis, using antibodies against the SBP tag and antibodies against LeishIF4E-3 (Fig. S1B). Since the added-back gene was introduced via an episomal vector, it was expressed at least 5-fold more highly than the endogenous gene.

### Reduced LeishIF4E-3 expression affects parasite growth and global translation rates.

**FIG 2:**
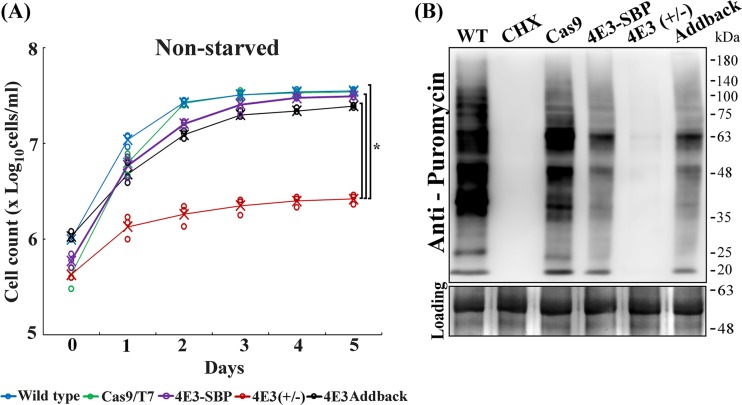
Reduced LeishIF4E-3 expression affects parasite growth and global translation. Wild-type L. mexicana, the transgenic parasite expressing Cas9/T7, the LeishIF4E-3(+/−) deletion mutant, and LeishIF4E-3 addback promastigotes were grown in DMEM containing all supplements in control experiments. (A) Cell growth was monitored by cell count using a Neubauer hemocytometer for 5 days. Wild-type cells are shown in blue, Cas9/T7-expressing cells are shown in green, LeishIF4E-3-SBP cells (expressing LeishIF4E-3 tagged with the streptavidin binding peptide [SBP]) are shown in purple, LeishIF4E-3(+/−) deletion mutant cells are shown in red, and LeishIF4E-3 addback cells are shown in black. Cell growth is represented by log_10_ cells per milliliter against number of days as dot plot. Cell counting was repeated three times. Lines were drawn based on the mean values of the different experiments and are marked with X’s. *P* values below 0.05 are marked with an asterisk. (B) All cell lines were incubated with 1 μg/ml puromycin for 20 min. A cycloheximide (CHX) control for translation inhibition is shown as a control for complete inhibition of translation. Whole-cell extracts (similar protein loads) were separated by 12% SDS-PAGE and subjected to Western analysis using antibodies against puromycin. The bottom lane of Ponceau staining shows the protein loads. The experiment was repeated three times.

We evaluated the effect of the LeishIF4E-3(+/−) deletion on promastigote growth. Figure 2A shows a severe growth defect of the LeishIF4E-3(+/−) heterologous mutant cells under normal conditions, compared to wild-type or Cas9/T7-expressing promastigotes. The growth defect was repaired in the addback cell lines, in which expression of LeishIF4E-3 was restored (Fig. 2A). We also monitored the cell growth under purine deprivation conditions, showing that removal of purines from the growth medium impaired the growth of all cell lines but that the growth of the LeishIF4E-3 heterologous deletion mutant, LeishIF4E-3(+/−), was slower (Fig. S2A).

10.1128/mSphere.00450-19.3 FIG S2 Reduced expression of LeishIF4E-3 affects parasite growth and global translation. (A) Growth of LeishIF4E-3(+/−) deletion mutant, parasites expressing SBP-tagged LeishIF4E-3, addback parasites, the transgenic line expressing Cas9/T7, and wild-type L. mexicana was monitored under purine starvation conditions for 5 days. Wild-type cells are shown in blue, Cas9/T7-expressing cells are shown in green, LeishIF4E-3-SBP cells are shown in purple, LeishIF4E-3(+/−) heterologous mutant cells are shown in red, and LeishIF4E-3 addbacks are shown in black. Cell growth is represented by log_10_ cells per milliliter against number of days. Mean growth values are presented as a line denoted by X’s. (B) (I) High contrast of the Western blot for global translation as shown in Fig. 2B. (II) Densitometric analysis of global translation under normal conditions was performed on each lane of the Western blot shown in Fig. 2B. Each lane was fully quantified using the Multi Gauge software, version 2.0. The values were normalized to the protein loads and presented as dot plots. (C) Cellular metabolism in LeishIF4E-3(+/−) deletion mutant and wild-type cells was determined by XTT assay. Metabolism was followed by measuring the ability of metabolically active cells to reduce XTT to orange formazan salt. The absorbance of the color produced in each sample was measured against a background control as a blank at a wavelength of 450 nm. Reference absorbance was measured at a wavelength of 630 nm and subtracted from the absorbance at 450 nm. The experiment was repeated six times, and the measured OD_450-630_ for the LeishIF4E-3(+/−) deletion mutant and for wild-type cells is presented as mean with SD. Statistical significance is shown by *P* < 0.05 and noted by an asterisk. Download FIG S2, PDF file, 0.4 MB. Copyright © 2019 Shrivastava et al. 2019 Shrivastava et al. This content is distributed under the terms of the Creative Commons Attribution 4.0 International license.

Reduced rates of proliferation are expected to correlate with reduced translation rates. The effect of the LeishIF4E-3(+/−) deletion on global translation was followed using the surface sensing of translation (SUnSET) assay. This assay is based on the incorporation of puromycin, a known structural analogue of tRNA, into the A site of the ribosome and hence into the growing polypeptide chain. Incorporation of puromycin stops the elongation of the growing polypeptide chain, generating puromycin-tagged polypeptide chains. These tagged chains can be further detected over Western blot assays using antibodies that target puromycin (21). Figure 2B shows that ectopically expressed genes could partially slow down global translation, but this did not have a dramatic effect on growth rates. However, the reduced global translation in the LeishIF4E-3(+/−) mutant was more severe and resulted in slower growth, although the cells remained viable throughout the period in which growth was monitored (5 days). Cells that were seeded at a concentration of 5 × 10^5^ cells/ml multiplied only twice, reaching a final concentration of 2 × 10^6^ cells/ml after 5 days. A parallel inhibitory effect was observed on the total metabolic activity, which was reduced by 42% (Fig. S2C). As in metacyclogenesis, *Leishmania* cells that reduce translation and metabolism do not lose their viability for prolonged time periods. All Western blots were quantified by densitometry, and cells treated with cycloheximide served as control for a complete arrest of translation (Fig. S2B).

In addition, we monitored the effect of the LeishIF4E-3(+/−) deletion on cellular metabolism by the XTT [2,3-bis-(2-methoxy-4-nitro-5-sulfophenyl)-2H-tetrazolium-5-carboxanilide] assay. This colorimetric assay is based on the reduction of XTT by the mitochondrial enzymes of living cells, to produce colored formazan compounds (22). Figure S2C shows a decline in the mitochondrial activity of the LeishIF4E-3(+/−) deletion mutant cells, with a reduction of 42% compared to the observed absorbance measured in wild-type cells. The reduced metabolism could also be a reason for the decrease in translation, possibly indirectly.

### The LeishIF4E-3(+/−) mutant cells form LeishIF4E-3 containing granules under all nutritional conditions.

**FIG 3:**
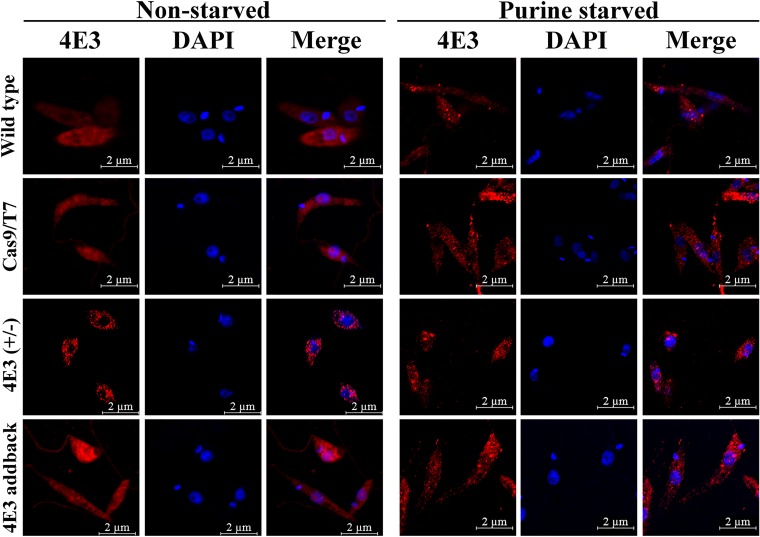
The mutant LeishIF4E-3(+/−) cells form LeishIF4E-3-containing granules under all nutritional conditions. Wild-type L. mexicana, the transgenic parasite expressing Cas9/T7, LeishIF4E-3(+/−) deletion mutants, and LeishIF4E-3 addback promastigotes were subjected to purine starvation for 4 days (right panel, purine starved). Parallel control cultures were maintained under normal conditions with complete supplements (left panel, nonstarved). All cells were fixed, permeabilized, and processed for confocal microscopy. LeishIF4E-3 was detected using specific rabbit anti-LeishIF4E-3 antibodies and secondary DyLight-labeled antibodies (550 nm; red). Nuclear and kinetoplast DNA was stained using DAPI (blue). A bright-field (BF) picture of the cells is on the right. The confocal analysis was repeated three times.

Reduced global translation often correlates with the assembly of cytoplasmic stress granules that have variable storage functions (16). To understand the role played by LeishIF4E-3 in assembly of these granules, we used the LeishIF4E-3(+/−) deletion mutant and tested its ability to generate such granules under normal conditions and in response to a nutritional stress. Although granule formation in response to nutritional stress was observed in all the cell lines [including the LeishIF4E-3(+/−) mutant], this was not the case under normal conditions. Under these conditions, only the LeishIF4E-3(+/−) deletion mutant generated LeishIF4E-3-containing granules (Fig. 3 and Fig. S3). Granule formation is typically triggered by the reduction in translation, when the cells must store or degrade their inactive ribosomes and transcripts. Thus, if LeishIF4E-3 expression was reduced, as observed in the mutant cells, the formation of storage granules was expected (Fig. 3 and Fig. S3).

10.1128/mSphere.00450-19.4 FIG S3 Field view showing granule formation in the mutant LeishIF4E-3(+/−) parasites. LeishIF4E-3(+/−) deletion mutant cells, LeishIF4E-3 addbacks, transgenic parasites expressing Cas9/T7, and wild type L. mexicana cells were subjected to purine starvation for 4 days. Control cells were grown in parallel under normal conditions. LeishIF4E-3 was detected using specific rabbit anti-LeishIF4E-3 antibodies and secondary DyLight-labeled antibodies (550 nm; red). Nuclear and kinetoplast DNA was stained using DAPI (blue). A bright-field (BF) picture of the cells is on the right. Download FIG S3, PDF file, 0.3 MB. Copyright © 2019 Shrivastava et al. 2019 Shrivastava et al. This content is distributed under the terms of the Creative Commons Attribution 4.0 International license.

### The LeishIF4E-3(+/−) deletion mutant shows altered morphology under all nutritional conditions.

**FIG 4:**
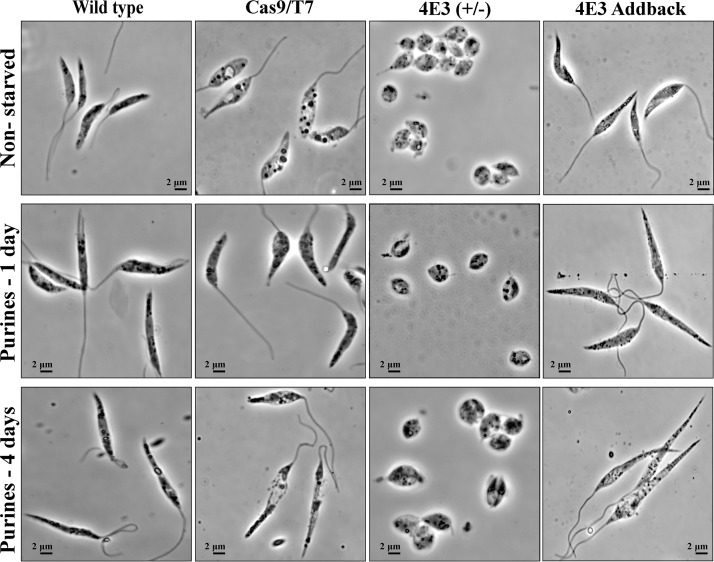
LeishIF4E-3(+/−) cells do not transform into nectomonad-like forms. LeishIF4E-3(+/−) cells along with control wild-type cells, Cas9/T7-expressing cells, and addback cells were starved for purines for 1 to 4 days. Controls were grown under normal conditions (nonstarved). The cells were fixed and visualized by phase-contrast microscopy at ×100 magnification. Under normal conditions, the LeishIF4E-3(+/−) mutant cells are round and equipped with a short flagellum, unlike control wild-type cells, cells expressing Cas9/T7, or the addback cells in which expression of LeishIF4E-3 was recovered. Furthermore, purine starvation generated nectomonad-like cell forms only in the three control lines, but the LeishIF4E-3(+/−) deletion cells maintained their rounded cell shape with the short flagellum even under prolonged purine starvation conditions of 4 days. The presented images are representative of three individual repeats.

Previously, we showed that in response to nutrient stress, especially elimination of purines, wild-type *Leishmania* cells go through a morphological change that turns them into highly motile nectomonad-like cells, which are elongated and equipped with a long flagellum (16). The LeishIF4E-3(+/−) mutant cells, along with control wild-type cells, Cas9/T7 expressers, and LeishIF4E-3 addback cells, were starved for purines during 4 days, and parallel control cultures were not starved. The cell morphology of all strains under both conditions was analyzed by phase-contrast microscopy. Figure 4 shows that reduced expression of LeishIF4E-3 led to altered promastigote morphology under normal conditions. These cells exhibited a rounded shape and a short flagellum, whereas control wild-type or LeishIF4E-3 addback cells had an elongated shape with a normally protruding flagellum. The morphological changes were even more pronounced when the cells were exposed to purine starvation. While wild-type and Cas9/T7 control cells obtained a nectomonad-like cell shape, i.e., long, slender, and equipped with a longer flagellum, the LeishIF4E-3(+/−) deletion mutant cells maintained their rounded shape and short flagellum. We relate the failure to induce the typical morphological transformation to the low expression of LeishIF4E-3 in the heterologous deletion mutant since restored expression of LeishIF4E-3 in the addback cells let them regain their ability to form nectomonad-like cells in response to purine starvation.

**FIG 5:**
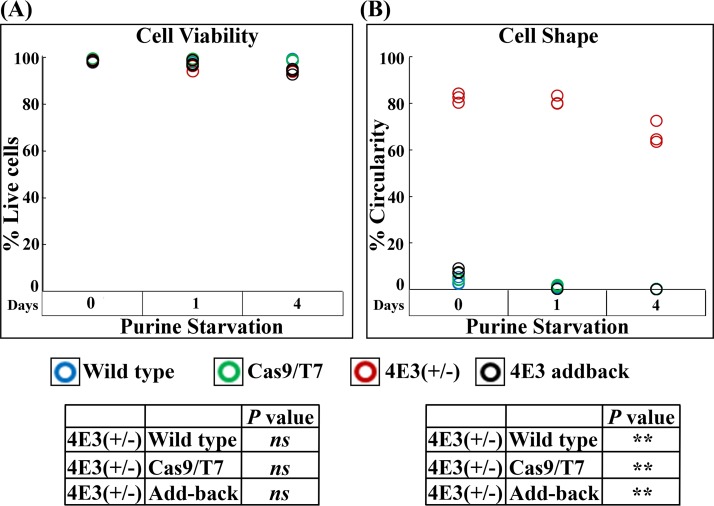
Flow cytometry analysis showing cell shape and viability of LeishIF4E-3(+/−) mutant cells in response to purine starvation. LeishIF4E-3(+/−) cells along with control wild-type cells, Cas9/T7-expressing cells, and addback cells were starved for purines for 1 to 4 days. Controls were grown under normal conditions (nonstarved). Using flow cytometry, we measured the circularity and elongatedness of single, viable, and focused cells of all the strains. Percentage of viable cells (A) and percentage of rounded cells in the population (B) are shown as dot plots. Flow cytometry analysis data were obtained from three independent repeats. Statistical differences in the individual values are shown in the table. A *P* value below 0.01 is marked by a double asterisk, and “ns” represents nonsignificant differences.

Changes in cell shape in response to purine starvation were also analyzed using flow cytometry. We first verified that purine depletion did not affect cell viability (Fig. 5A and Fig. S4A) (also as previously published [16]). Flow cytometry analysis confirmed that ∼81% of the LeishIF4E-3(+/−) mutant cells were rounded, compared to ∼4% of cells in wild-type and in Cas9/T7-expressing cells, or 7.98% in the LeishIF4E-3 addback cells (Fig. 5B and Fig. S4B and C). Flow cytometry analysis confirmed that the majority of the LeishIF4E-3(+/−) parasites were rounded even under normal conditions and did not show a change following purine deprivation during 24 h (∼80%) or even 4 days (65%, Fig. 4 and Fig. 5B).

10.1128/mSphere.00450-19.5 FIG S4 Flow cytometry analysis of LeishIF4E-3(+/−) mutant morphology under normal conditions and in response to purine starvation. LeishIF4E-3(+/−) deletion mutant cells, LeishIF4E-3 addbacks, transgenic parasites expressing Cas9/T7, and wild-type L. mexicana cells were starved for purines for 24 h or 4 days. Control lines were grown under normal conditions. The cells (10^7^) were stained with propidium iodide (PI) for 30 min. Viability, circularity, and cell length of 20,000 cells were recorded with an Image Stream X Mark II flow cytometer. (A) Graphs representing cell viability for focused, single gated cells are shown for all the treatments (red). The flow cytometry pattern of live and dead cells, with and without PI, is shown at the bottom (green). (B) Scatter plots representing gated focused single cell populations are shown for all the treatments. (C) Gated cell populations representing the circular or elongated cell shapes are shown as scatter plots. Download FIG S4, PDF file, 0.6 MB. Copyright © 2019 Shrivastava et al. 2019 Shrivastava et al. This content is distributed under the terms of the Creative Commons Attribution 4.0 International license.

### The LeishIF4E-3(+/−) deletion mutant shows reduced infectivity in murine macrophages.

**FIG 6:**
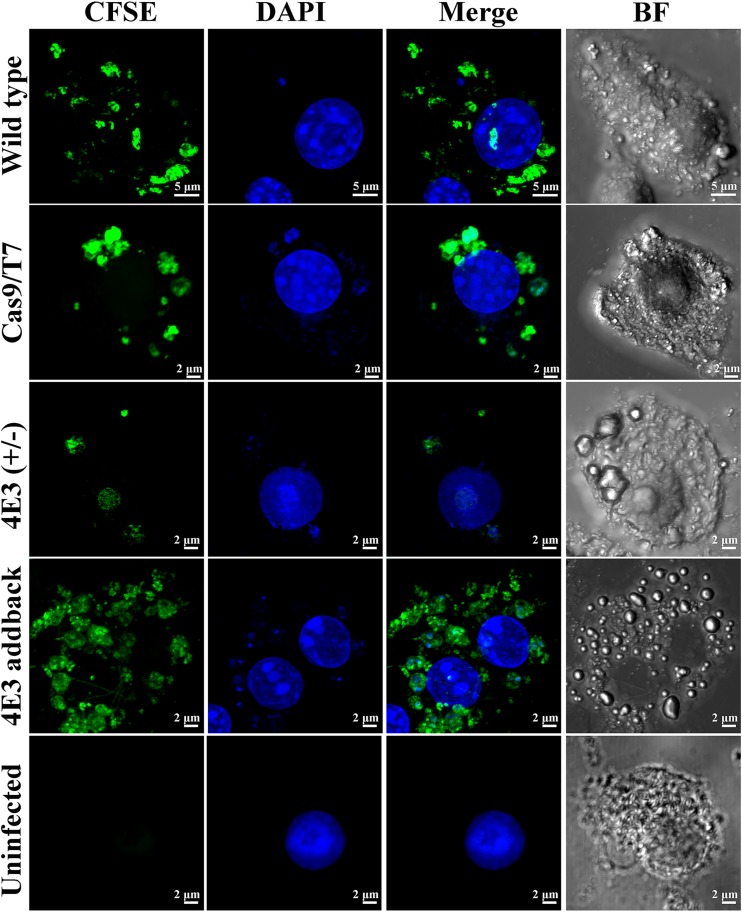
LeishIF4E-3(+/−) deletion mutants show reduced infectivity of cultured macrophages. The LeishIF4E-3(+/−) deletion mutant and LeishIF4E-3 addback cells, along with wild-type L. mexicana and transgenic parasites expressing Cas9/T7, were grown in DMEM containing all supplements for 5 days. The parasites were stained with CFSE and allowed to infect the RAW 264.7 macrophage cells for 1 h. The macrophages were washed and further incubated in supplemented DMEM. Parasite infectivity of macrophages was monitored 24 h postinfection by confocal microscopy. The CFSE-stained *Leishmania* cells are stained in green, while DAPI stained the nuclear material of macrophages and *Leishmania* (blue). A bright-field (BF) picture of the cells is on the right. A representative image is presented for macrophage infection by each cell line derived from three independent repeats.

The long nectomonad forms are precursors of metacyclic promastigotes, which are the virulent life form of *Leishmania* promastigotes (23). We therefore examined the infectivity of the LeishIF4E-3(+/−) mutant cells by their ability to infect cultured RAW 264.7 macrophages, using confocal microscopy. *Leishmania* parasites were stained with carboxyfluorescein succinimidyl ester (CFSE) (green), as observed in Fig. S5A and B, and 4′,6-diamidino-2-phenylindole (DAPI) was used to stain DNA (blue). The ability to infect macrophages was measured by counting the number of infected cells following 24 h, in fields that contained a total of 100 macrophages. The results (Fig. 6 and Fig. S5C) show that the control wild-type and Cas9/T7 cells infected 84.6% ± 9.8% and 80.4% ± 8.43% of the macrophage cells, respectively, whereas the LeishIF4E-3(+/−) deletion mutant could infect only 49.7% ± 7.75% of the cells. The addback parasite cells recovered their infectivity, infecting 84.4% ± 9.89% of the macrophage cells (Table 1). The infectivity of the different lines was also monitored 4 h after infection (Fig. S5D and E). Wild-type and Cas9/T7 cells infected 95.8% ± 5.4% and 87.5% ± 6.6% of the macrophages, respectively, whereas the LeishIF4E-3(+/−) cells infected only 39.3% ± 10.1% of the cells. The addback mutant cells recovered their ability to infect the macrophage cells (96.3% ± 4.8%) (Table 1).

**TABLE 1:** Reduced macrophage infectivity of LeishIF4E-3(+/−) mutant^*a*^

Parameter	Cell line	Mean	SD	*P* value^*d*^
% infected cells^*b*^				
24 h	WT	84.60	9.80	***
	Cas9/T7	80.39	8.43	**
	4E-3(+/−)	49.73	7.75	
	Addback	84.41	9.89	**
4 h	WT	95.79	5.43	**
	Cas9/T7	87.50	6.66	**
	4E-3(+/−)	39.32	10.15	
	Addback	96.30	4.79	**
No. of parasites per infected cell^*c*^				
4 h	WT	4.68	1.50	**
	Cas9/T7	3.95	1.06	**
	4E-3(+/−)	1.58	0.40	
	Addback	4.53	1.16	**
24 h	WT	12.60	2.82	***
	Cas9/T7	11.90	1.07	***
	4E-3(+/−)	2.14	0.28	
	Addback	13.96	4.39	***

a The ability of the LeishIF4E-3(+/−) mutant to infect the macrophage RAW 264.7 cells was quantified by counting the number of infected macrophages. Control infections included wild-type parasites, the Cas9/T7-expressing cells, and the LeishIF4E-3 addback cells, in which expression of LeishIF4E-3 was recovered.

b Infectivity of each cell line was monitored by confocal microscopy as described in the legend to Fig. 6, at 24 and 4 h postinfection.

c Parasite loads in infected macrophages were also determined, by counting the number of parasites per infected macrophage. The cells were counted using a cell counting plugin of Image J. A total of 100 macrophages were counted three times for each parasite cell line.

d The statistical analysis for percentage of infected cells and average parasite per cell, along with standard deviation values (SD), was performed by Kruskal-Wallis test using GraphPad Prism. The number of infected macrophages and average number of parasite counts per infected macrophage were the two parameters that were compared between LeishIF4E-3(+/−) deletion mutants and each of the control lines: wild type, transgenic cells expressing Cas9/T7, and LeishIF4E-3 addback cells. ***, *P* < 0.001; **, *P* < 0.01. The statistical difference between the control lines was nonsignificant.

10.1128/mSphere.00450-19.6 FIG S5 The ability of LeishIF4E-3(+/−) deletion mutants to infect murine macrophage cells was monitored by confocal microscopy. LeishIF4E-3(+/−) deletion mutant and LeishIF4E-3 addback cells along with wild-type L. mexicana or the transgenic parasite expressing Cas9/T7 were grown in DMEM containing all supplements for 5 days. (A) Parasites from each cell line were stained with CFSE (green), enabling their visualization. Nuclear and kinetoplast DNA was stained using DAPI (blue). A bright-field (BF) picture of the cells is shown on the right. (B) Field view of cells shown in panel A. (C) Parasite infectivity: The CFSE-stained parasites were incubated with macrophages at a multiplicity of 10:1 parasites per macrophage, for 1 hour. Following infection, macrophages were washed 3 times with PBS, and infection of the macrophages was monitored 24 h postinfection. Figure 6 shows individual infected cells, and the broad field is presented here. (D) Infection of individual cells was also followed 4 h postinfection, with panel E showing the broad field. A bright-field (BF) picture of the cells is on the right. Download FIG S5, PDF file, 1.5 MB. Copyright © 2019 Shrivastava et al. 2019 Shrivastava et al. This content is distributed under the terms of the Creative Commons Attribution 4.0 International license.

The reduced infectivity was also evaluated by counting the average number of parasites per infected cell, at 4 and 24 h following infection. Parasite counts per infected cell of wild-type cells, Cas9/T7 expressers, and addback controls were 4.7 ± 1.5, 3.95 ± 1.05, and 4.53 ± 1.16, respectively, after 4 h of infection. However, macrophages infected by the LeishIF4E-3(+/−) deletion mutant contained only 1.6 ± 0.4 parasites per cell after 4 h (Table 1). When the infection was measured after 24 h, the macrophages infected by wild type, Cas9/T7 expressers, and addback controls contained 12.6 ± 2.8, 11.92 ± 1.07, and 13.96 ± 4.4 parasites per cell, respectively. However, macrophages infected with the LeishIF4E-3(+/−) mutant contained only 2.14 ± 0.6 parasites per infected cell (Table 1). The differences in parasite numbers per infected cell were significant.

### RNA-Seq analysis of LeishIF4E-3-associated RNAs.

**FIG 7:**
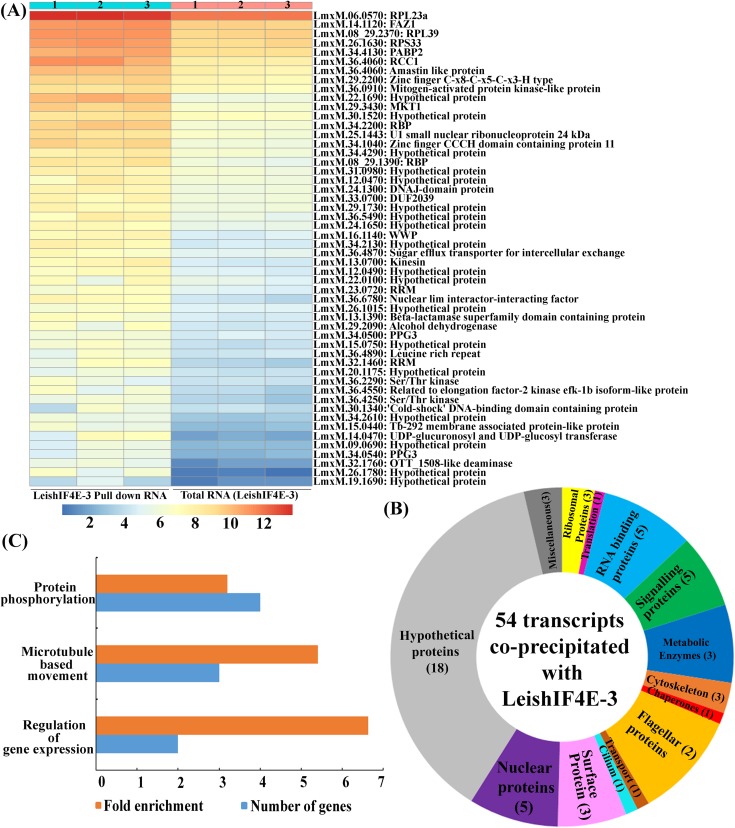
RNA-Seq analysis of LeishIF4E-3 associated transcripts. SBP-tagged LeishIF4E-3 was enriched over streptavidin-Sepharose beads, and the associated RNAs were extracted and further subjected to RNA-Seq analysis, in triplicates. Total mRNAs enriched over a poly(dT) column served as control. Enrichment of specific transcripts in the pulled-down fraction was determined by the DESeq2 tool, and the statistical significance was based on the Wald test. (A) A heat map showing the log_2_ of normalized read counts for each enriched transcript in the LeishIF4E-3 pulled-down fraction, compared to the pattern observed in total RNA. The different columns show the three biological repeats of the pulled-down and total RNAs; the rows represent the individual gene transcripts. The color scale ranging from blue to red demonstrates the log_2_ count numbers. (B) A pie chart representation of transcripts that were enriched by 3-fold with an adjusted *P* value of <0.05. The transcripts were manually categorized according to their function. The different slices in the pie represent the summed log_2_ fold change of each transcript in the assigned category. The numbers in brackets in the pie chart represent the number of genes in each category. Transcripts annotated to hypothetical proteins and proteins with nondefined functions are in gray, while transcripts with known molecular functions are differently colored. (C) GO term enrichment of differentially expressed genes by biological processes. The resulting GO terms were enriched by 3-fold compared to the gene sets contained in the genome, with a *P* value of <0.01.

Given the reported involvement of LeishIF4E-3 in global translation and the effect of its reduced expression on cell morphology and flagellum growth, we analyzed the profile of transcripts that associate with LeishIF4E-3, using RNA coprecipitation experiments. Transgenic parasites expressing SBP-tagged LeishIF4E-3 were affinity purified over streptavidin-Sepharose beads. The tagged LeishIF4E-3 was eluted by biotin, and its associated transcripts were extracted and subjected to RNA sequencing (RNA-Seq) analysis. Total cellular RNA that was extracted from the same transgenic cells and subjected to poly(A) enrichment was used as control. Attempts to generate RNA libraries from putative control cells expressing SBP-tagged luciferase did not succeed, since they did not generate sufficient amounts of RNA to allow library preparation and subsequent RNA-Seq analysis. An alternative approach, of comparing the transcripts pulled down via the SBP-tagged LeishIF4E-3 with transcripts pulled down from the LeishIF4E-3(+/−) deletion mutant, was also not feasible, since the LeishIF4E-3 in the CRISPR-Cas9-derived mutant cells was untagged, preventing its pulldown via SBP. We were also prevented from generating RNA libraries from the LeishIF4E-3(+/−) cells, since the target gene was not endogenously tagged. Overall, 56 to 72 million reads for the total and LeishIF4E-3-associated transcripts were generated. The log_2_ of normalized read counts for each transcript that was enriched in the LeishIF4E-3-associated fraction and in the total control sample is shown for each biological repeat as a heat map (Fig. 7A, based on Table S1). Transcripts that showed a 3-fold enrichment of the LeishIF4E-3-associated fraction, compared to controls, with an adjusted *P* value of <0.05 were manually categorized into functional groups (Fig. 7B and Table S1). The manual classification highlighted the enrichment of transcripts encoding flagellar proteins, several RNA binding proteins, and ribosomal proteins, as well as transcripts coding for signaling, chaperone, cytoskeletal, and surface proteins. A large portion of the transcripts that were enriched in the LeishIF4E-3 fraction were nonannotated, as they are unique to trypanosomatids and have no apparent orthologs in other organisms. The genes were also analyzed for their Gene Ontology (GO) term enrichment for their metabolic processes, using a threshold of 3-fold enrichment compared to the gene sets contained in the genome, with a *P* value of <0.01 (Fig. 7C and Table S2). The GO term analysis also highlighted the enrichment of proteins involved in gene expression regulation, protein phosphorylation, and microtubule-based movement.

10.1128/mSphere.00450-19.7 TABLE S1 Identified transcripts found enriched with LeishIF4E-3—manual categorization. The LeishIF4E-3-associated transcripts were enriched by pulldown analysis over streptavidin-Sepharose beads. The transcripts were subjected to RNA-Seq analysis, and the identified transcripts were compared to the transcripts identified in total RNA from SBP-tagged LeishIF4E-3-expressing cells. Total mRNAs enriched over a poly(dT) column served as control. Enrichment of specific transcripts in the pulled-down fraction was determined by the DESeq2 tool, and the statistical significance was based on the Wald test. All identified transcripts were enriched by a log_2_ fold change of 3, with an adjusted *P* value of <0.05, and are shown on sheet 1. DESeq2-normalized read counts were log_2_ transformed for each transcript obtained from LeishIF4E-3 pulled-down fraction and from total RNA obtained from transgenic cells expressing SBP-tagged LeishIF4E-3 and are shown in sheet 2. Download Table S1, XLSX file, 0.7 MB. Copyright © 2019 Shrivastava et al. 2019 Shrivastava et al. This content is distributed under the terms of the Creative Commons Attribution 4.0 International license.

10.1128/mSphere.00450-19.8 TABLE S2 Identified transcripts found enriched with LeishIF4E-3—classification by GO term enrichment. The LeishIF4E-3-associated transcripts were enriched by pulldown analysis over streptavidin-Sepharose beads and were subjected to RNA-Seq analysis. The transcripts that were found enriched with LeishIF4E-3 are given in Table S1 and were analyzed by the GO term enrichment tool based on biological processes. All GO terms were enriched by at least 3-fold compared to the gene sets contained in the genome, with a *P* value of <0.01. Download Table S2, XLSX file, 0.01 MB. Copyright © 2019 Shrivastava et al. 2019 Shrivastava et al. This content is distributed under the terms of the Creative Commons Attribution 4.0 International license.

## DISCUSSION

The cap-binding affinities of the six identified eIF4E orthologs in trypanosomatids vary (13, 24) along the life cycle (25). These multiple eIF4E orthologs are therefore proposed to perform specialized stage-specific functions that may not be exclusively related to protein synthesis. The use of RNAi in trypanosomes showed that T. brucei eIF4E-3 (TbIF4E-3) is essential and was recently suggested to have a role in the regulation of translation (26). The development of the CRISPR-Cas9 system for *Leishmania* (27) enables us now to address similar questions in *Leishmania*.

Our attempts to eliminate the two LeishIF4E-3 alleles resulted in the replacement of only a single copy. The mutant LeishIF4E-3(+/−) cells were viable, but expression of the target protein was reduced and the phenotype of the cells was altered, affecting parasite growth, translation, morphology, and infectivity. LeishIF4E-3 expression level therefore appears to be central to various cellular functions of the parasite.

The weak affinity of LeishIF4E-3 was attributed to alteration of one of the three conserved tryptophan residues in the cap-binding pocket (13) to methionine. With respect to its binding affinities, it should be emphasized that these were determined with recombinant proteins that were not subject to posttranslational modifications (13). Binding of the endogenous protein also seems to vary between the different cap-binding proteins, and we assume that specific auxiliary proteins that participate in the cap-binding complex could be involved in regulating the cap-binding activity. RNA binding proteins that are part of the cap-binding complex could be involved in enhancing translation mediated by LeishIF4E-3 *in vivo*.

A decline in translation was observed in most cell lines that expressed any gene ectopically, from an episomal expression vector. This was observed in cells expressing the Cas9/T7 genes, as well as in cell lines expressing the tagged LeishIF4E-3. This decline could be due to competition over a given pool of translation factors or chaperones that accompany translating ribosomes. However, the reduced expression of LeishIF4E-3 in the deletion mutant led to a much stronger decline in translation than in the control cells. It should also be noted that elimination of one LeishIF4E-3 allele resulted in reduced cellular metabolism, which could lead to reduced translation and the formation of LeishIF4E-3-containing granules, even under full nutritional conditions. Thus, LeishIF4E-3 could be involved in translation regulation, although the exact mode is not yet understood.

The reduction in LeishIF4E-3 expression resulted in altered morphology of promastigotes, leading to a round cell shape and a short flagellum. Moreover, the LeishIF4E-3(+/−) mutant cells failed to change their morphology in response to purine availability, as previously reported for wild-type cells (16). Upon exposure to purine deprivation, *Leishmania* typically undergoes a morphological transition to nectomonad-like cells, which are long, slender, and equipped with an elongated flagellum. However, the LeishIF4E-3(+/−) cells lost their ability to transform into the nectomonad-like cells in response to purine starvation. When LeishIF4E-3 expression was restored (i.e., in the addback cells), the cells resumed the morphology which is typical of wild-type promastigotes and recovered their ability to respond to the purine starvation by forming the nectomonad-like cells. Thus, it appears that there is a threshold level of LeishIF4E-3 expression that is required for its function under normal conditions. When its level goes down below the required threshold, translation slows down and becomes imbalanced with the mRNA and ribosome pools in the cell. As a result, the cell targets them to storage granules along with LeishIF4E-3, even under normal conditions, as observed in Fig. 3.

The LeishIF4E-3(+/−) mutant cells also showed reduced infectivity in a macrophage cell line, as monitored by counting the percentage of infected macrophages and by calculating the average number of parasites per infected cell. These results were obtained at two time points, 4 and 24 h postinfection, enabling us to distinguish between parasite entry (measured after 4 h), and parasite survival within the macrophages (measured after 24 h). A possible explanation for the reduced infectivity emanates from the fact that the LeishIF4E-3(+/−) mutant cells are equipped with a short flagellum and are severely restricted in their motility. This observation is in line with previous reports that highlight the role of the flagellum in promastigote entry into macrophages (28).

There is growing evidence implicating the role of *Leishmania* flagellum in macrophage invasion and infectivity (3, 28). In addition to the flagellum role in cellular movement, the flagellar membrane has been shown to play a key role in sensing the environment and generating signal transduction pathways (29). Similar reports were published for T. brucei, in which a subset of proteins are exclusively found at the tip and could play a role in the initiation of signaling events leading to the successful internalization and differentiation of parasites (30, 31). Different reports vary with respect to the polarity of parasite invasion, but the involvement of the flagellum in the entry process is rather established (32–34). The importance of flagellar pocket and flagellar attachment zone (FAZ) proteins has been extensively examined for their role in host infection. FAZ1 is of profound interest since its transcript was enriched in the fraction that associated with LeishIF4E-3. FAZ is a complex cytoskeletal structure, which influences cell morphology by regulating cell length and organelle position and is therefore essential for cell division. In T. brucei, knockdown of FAZ1 resulted in a compromised FAZ structure and generated cells with fully or partially detached flagella that were defective in cytokinesis (35). Mutants in the FAZ5 proteins also showed a dramatic reduction in parasite pathogenicity as measured in mice (28).

Transcripts that associate with LeishIF4E-3 were identified in RNA coprecipitation and RNA sequencing. This analysis revealed ∼54 transcripts enriched in the fraction that was precipitated by LeishIF4E-3. The enrichment of transcripts encoding cytoskeletal, surface, and flagellar proteins (FAZ1) is in agreement with our results that highlight morphological defects in the LeishIF4E-3(+/−) mutant, in which expression of LeishIF4E-3 is reduced. These defects reflect changes in the ability to grow a long flagellum. In addition, it appears that LeishIF4E-3 also associates with a variety of transcripts that encode metabolic enzymes. This is also reflected in the decrease in cellular metabolism, which could also be directly related to the defects in translation and in cell morphology. Overall, our data are in line with the possibility that LeishIF4E-3 could have a role in the synthesis of specific proteins under normal physiological conditions and an additional role in the storage of transcripts and ribosomal particles during conditions of nutritional deprivation.

## MATERIALS AND METHODS

### Cell culture.

Leishmania mexicana cells were cultured in M199 medium or in Dulbecco’s modified Eagle’s medium (DMEM), supplemented with 10% heat-inactivated fetal calf serum (FCS; Biological Industries), 4 mM l-glutamine, 0.1 mM adenine, 5 μg/ml hemin, 40 mM HEPES, pH 7.4, 100 U/ml penicillin, and 100 μg/ml streptomycin. Nonstarved control cells were grown in complete DMEM, fully supplemented as described above. Starvation for purines was performed by incubation in DMEM, except that supplements excluded adenine and dialyzed FCS was used.

RAW 264.7 macrophage cells were grown at 37°C in the presence of 5% CO_2_, in DMEM supplemented with 10% FCS, 4 mM l-glutamine, 0.1 mM adenine, 40 mM HEPES, pH 7.4, 100 U/ml penicillin, and 100 μg/ml streptomycin.

### CRISPR-Cas9-mediated knockout.

The CRISPR system plasmids for *Leishmania* were obtained from Eva Gluenz (University of Oxford, United Kingdom) (27). The pTB007 plasmid containing the gene encoding the Streptococcus pyogenes CRISPR-associated protein 9 (Cas9) endonuclease and the T7 RNA polymerase gene (Cas9/T7) along with the hygromycin resistance gene was transfected into L. mexicana cells. Stably transfected transgenic cells expressing Cas9 and T7 RNA polymerase were selected for hygromycin resistance (200 μg/ml).

**(i) Generation of LeishIF4E-3 deletion mutant by CRISPR-Cas9.** To generate LeishIF4E-3 null mutants, we used three PCR-amplified products: the 5′ and 3′ sgRNAs designed to create double-strand breaks upstream and downstream of the LeishIF4E-3 coding region and the LeishIF4E-3 repair cassette fragment containing the drug resistance markers (the neomycin or blasticidin resistance genes) as described in Text S1 in the supplemental material. The three PCR products were transfected into mid-log-phase transgenic cells expressing Cas9 and T7 RNA polymerase that were further subjected to selection with 200 μg/ml G418 and 20 μg/ml blasticidin hydrochloride (36).

10.1128/mSphere.00450-19.1 TEXT S1 Supplemental methods. Download Text S1, DOCX file, 0.03 MB. Copyright © 2019 Shrivastava et al. 2019 Shrivastava et al. This content is distributed under the terms of the Creative Commons Attribution 4.0 International license.

The sgRNA primers used to knock out the LeishIF4E-3 gene were originally designed using the EuPaGDT CRISPR gRNA design tool and obtained from LeishGEdit.net (37). The sgRNA primers contained the highest-scoring 20-nucleotide (nt) guide RNA sequence within 105 bp upstream or downstream of the target gene. In addition, we performed a BLAST search of the sgRNA sequences in the L. mexicana genome in TriTrypDB and found that the sgRNAs were highly specific for LeishIF4E-3 (E value = 0.001 and 8e−5). Further, we performed a BLAST search of the drug resistance repair cassette that contained the homology sequence in the UTR of LeishIF4E-3, enabling recombination. The repair cassette showed an E value of 5e−9, suggesting very high specificity of the system.

**(ii) PCR amplification of sgRNA templates.** DNA fragments containing LeishIF4E-3-specific 5′ and 3′ guide RNAs for cleavage upstream and downstream of the LeishIF4E-3 target gene were generated. The template for this PCR consisted of two fragments, one containing the common sgRNA scaffold fragment (5′-AAAGCACCGACTCGGTGCCACTTTTTCAAGTTGATAACGGACTAGCCTTATTTTAACTTGCTATTTCTAGCTCTAAAAC-3′) and the other containing the T7 RNA polymerase promoter (lowercase letters) fused to the LeishIF4E-3 UTR sequences (uppercase letters) and a short sequence overlapping the scaffold fragment (lowercase letters). The two individual template fragments for targeting a double-strand break at the 5′ end of LeishIF4E-3 were 5′-gaaattaatacgactcactataggTTCCCTCTGTGCCTAAACGCgttttagagctagaaatagc-3′), and the template fragment targeting a double-strand break at the 3′ end was 5′-gaaattaatacgactcactataggAAGGAGGCGCGAACGACATAgttttagagctagaaatagc-3′). Each of these two fragments was annealed to the partially overlapping scaffold fragment and further amplified with two small primers derived from the T7 promoter (forward, 5′-TTAATACGACTCACTATAGG-3′) and the common scaffold fragment (reverse, 5′-GCACCGACTCGGTGCCACTT-3′). All PCR products were gel purified from agarose gels and heated at 94°C for 5 min before transfection.

**(iii) PCR amplification of the LeishIF4E-3 selection fragment.** A DNA fragment designed to repair the double-strand breaks surrounding the LeishIF4E-3 target gene was amplified by PCR. The LeishIF4E-3-specific primers were derived from the 5′ and 3′ endogenous UTR sequences upstream and downstream of the LeishIF4E-3 gene and the sequences from the antibiotic repair cassette, based on the LeishGEdit database (http://www.leishgedit.net/Home.html). The primers were 5′-CCAGTCACACGTGTGACCCCCCTTCCACCAgtataatgcagacctgctgc-3′ (forward) and 5′-CTTCTCGCGATCCTTCTTCCCTCGTCTCCCccaatttgagagacctgtgc-3′ (reverse). Uppercase and lowercase letters represent the UTR sequences and the antibiotic resistance gene, respectively. The PCR was performed using the pT Neo/Bla plasmid as the template, generating a fragment used for repair of the double-strand breaks on both sides of the gene targeted for deletion, enabling the subsequent integration of the drug resistance marker by homologous recombination at the target site.

**(iv) Diagnostic primers for PCR of the deletion cell line.** To screen for the deletion of LeishIF4E-3, the following primers were used: LeishIF4E-3 forward (5′-ATGAACCCGTCTGCCGCTGC-3′), LeishIF4E-3 reverse (5′-ACAGAAGGTGTGATCGGGC-3′), LeishIF4E-3 5′ UTR (5′-CTTTTCACCATCAAGTCTCGGC-3′), LeishIF4E-3 3′ UTR (5′-CACCACGTACTCCCCACACAC-3′), and G418 reverse (5′-TGGCCAGCCACGATAGCCGC-3′).

**(v) Diagnostic PCR of the deletion cell line.** To screen for the deletion of LeishIF4E-3, genomic DNA from the drug-resistant cells was isolated and analyzed for the presence of the LeishIF4E-3 gene using specific primers, derived from the open reading frame. A parallel reaction was performed with primers derived from the 5′ and 3′ UTRs of the LeishIF4E-3 gene. To verify the replacement of LeishIF4E-3 with the drug resistance gene, a PCR was performed using a reverse primer derived from the drug resistance gene and a forward primer derived from the 5′ UTR of LeishIF4E-3. Genomic DNA from Cas9/T7 L. mexicana cells was used as control. PCR products were separated over 1% agarose gels.

### Generation of LeishIF4E-3 addback parasites.

The transgenic LeishIF4E-3(+/−) deletion mutant cells were transfected with an episomal transfection vector that was based on pT-Puro that confers resistance to puromycin, and they contained the SBP-tagged LeishIF4E-3 from L. mexicana cloned between two intergenic regions derived from the HSP83 (H) genomic cluster. Stably transfected cells were selected for resistance to puromycin. The pT-Puro-LeishIF4E-3 plasmid was generated as follows. The open reading frame of LeishIF4E-3 from L. mexicana was amplified using the forward primer 5′-actggatccATGAACCCGTCTGCCGCAGC-3′ and reverse primer 5′-gctctagaACAGAAGGTGTGATCG-3′, with BamHI and XbaI sites introduced at the 5′ ends of these primers (lowercase letters). The BamHI/XbaI PCR product was cloned into the BamHI and XbaI sites of the pX-H-SBP-H expression cassette between two intergenic regions derived from the HSP83 genomic locus (38, 39). The fragment containing the SBP-tagged LeishIF4E-3 open reading frame (ORF) and the two HSP83 flanking intergenic regions was extracted by a HindIII cleavage, blunted, and cloned into the blunted SfoI site of pT-Puro (27). The resulting pT-Puro-LeishIF4E-3 expression vector was transfected into the heterologous LeishIF4E-3(+/−) deletion mutant, and cells were selected for their resistance to puromycin (50 μg/ml).

### Western analysis.

Cells were harvested, washed with phosphate-buffered saline (PBS) (pH 7.4), and washed once in postribosomal soup (PRS) buffer (35 mM HEPES, pH 7.5, 100 mM KCl, 10 mM MgCl_2_, 1 mM dithiothreitol [DTT]). The cell pellet was resuspended in PRS buffer that was supplemented with a 2× cocktail of protease inhibitors (Sigma) and 4 mM iodoacetamide (Sigma), along with the phosphatase inhibitors sodium fluoride (25 mM), β-glycerophosphate (55 mM), and sodium orthovanadate (5 mM) (PRS^+^). Cells were lysed in Laemmli sample buffer and heated at 95°C for 5 min. Cell extracts were resolved on 12% SDS-polyacrylamide (SDS-PAGE) gels and probed using specific antibodies against LeishIF4E-3 and its binding partner LeishIF4G-4 along with antibodies against the streptavidin binding protein (SBP) tag. Equal protein loads were verified using specific antibodies against LeishIF4A-1. All the primary antibodies were used at 1:5,000 dilutions.

### Growth analysis.

Mid-log-phase LeishIF4E-3(+/−) deletion mutant and the addback L. mexicana cells were seeded at a density of 1 × 10^6^ cells/ml to analyze their growth. Wild-type L. mexicana cells and the transgenic cell lines expressing the Cas9/T7 genes served as controls. Cells were counted on a daily basis. Growth was also monitored under purine starvation conditions for 5 days, while control cells were grown in fully supplemented DMEM. Growth curves were performed in triplicates, with individual values shown in dot plots.

### Translation assay.

*De novo* translation was monitored by the SUnSET assay, which follows the incorporation of puromycin into the A site of the ribosome, tagging the nascent polypeptide chains, which are further monitored by Western blotting assays (21). Parasites were treated with 1 μg/ml of puromycin (Sigma) for 20 min, washed twice with ice-cold 1× PBS and once with PRS buffer, and finally resuspended in PRS^+^ buffer. The cell pellets were lysed in Laemmli sample buffer. A control of cycloheximide-treated wild-type parasites demonstrated a complete translation arrest. Cell extracts (equal protein loads) were resolved on 12% SDS-PAGE gels. Western analysis was performed using anti-puromycin monoclonal antibodies (Developmental Studies Hybridoma Bank, University of Iowa; 1:1,000) with horseradish peroxidase (HRP)-labeled anti-mouse secondary antibodies (KPL; 1:5,000).

### XTT assay.

Mid-log-phase LeishIF4E-3(+/−) deletion mutant and the wild-type control cells were seeded at a density of 1 × 10^5^ cells/ml in a 96-well plate and allowed to grow for 48 h in M199 medium without phenol red (Sigma). Cells were labeled with 2,3-bis-(2-methoxy-4-nitro-5-sulfophenyl)-2H-tetrazolium-5-carboxanilide (XTT), a tetrazolium-based compound, by addition of 50 μl reaction solution (50:1 XTT/activation solution ratio) per well. The XTT plate was incubated for 4 h at 25°C. Following incubation, absorbance of the samples was measured against a background control as a blank at a wavelength of 450 nm. Reference absorbance was measured at a wavelength of 650 nm and subtracted from the absorbance at 450 nm. The experiment was repeated six times, and the mean absorbance (*A*_450_ − *A*_650_) is presented as histogram with standard deviations.

### Phase-contrast microscopy of *Leishmania* promastigotes.

Mid-log-phase LeishIF4E-3(+/−) deletion mutant and addback L. mexicana cells were examined by phase-contrast microscopy. Control cells consisted of wild-type parasites or cells expressing Cas9/T7. The cells were exposed to purine deprivation for periods that ranged between 24 h and 4 days (see Text S1 in the supplemental material), along with nonstarved control cells. Cells were harvested, washed, fixed in 2% paraformaldehyde in PBS, and mounted. Phase-contrast images of parasites were captured at ×100 magnification with a Zeiss Axiovert 200M microscope equipped with an AxioCam HRm charge-coupled device (CCD) camera.

### Flow cytometry analysis of *Leishmania*.

LeishIF4E-3(+/−) deletion mutants and LeishIF4E-3 addback cells in their mid-log phase of growth were subjected to flow cytometry analysis, with wild-type and transgenic parasites expressing Cas9/T7 as controls. All lines were starved for purines, for defined time intervals; control lines were not starved. To examine viability under the different treatments, the cells were incubated with 20 μg/ml propidium iodide (PI) for 30 min. The stained cells were analyzed using an ImageStream X Mark II Imaging flow cytometer (Millipore) with a 60×/0.9 objective. Data from channels representing bright field as well as fluorescence (PI) emission at 617 nm (to evaluate cell viability) were recorded for 20,000 cells for each analyzed sample. IDEAS software generated the quantitative measurements of the focused, single, and live cells for all four examined cell strains. Cell shape was quantified using circularity and elongatedness features applied on the bright-field image processed by adaptive erode mask. Representative scatter plots are shown for focused single cells and for circularity (cell shape). Cell viability was measured by recording the emission of PI in the gated population.

For data analysis, IDEAS software was used to generate the quantitative measurements of the images recorded for the examined cell population. First, the focus quality of each cell was determined by measuring the gradient root mean square (RMS) value. The cells representing high value in the gradient RMS histogram were gated to select cells in focus. In the second step, single cell populations were gated from the scatter plot of aspect ratio/area to exclude cell aggregates. Further, the intensity of PI staining was used to exclude dead cells. The remaining living, single cells in focus were subjected to image analysis to determine cell morphology. To obtain cell shape, a customized adaptive erode mask was used on the bright-field channel, with a coefficient of 78. We further customized this mask to exclude the flagellum from the cell shape analysis. Further, circularity and elongatedness features were measured. A predetermined threshold value of 4 was set to define circularity. Elongatedness values represent the ratio between the cell length and width. Representative scatter plots are presented for focused single cells and for circularity. All data shown are from a minimum of three biological replicates.

### Confocal microscopy of *Leishmania* promastigotes.

For slide preparation, the cells were washed with PBS, fixed in 2% paraformaldehyde for 30 min, washed once with PBS, and allowed to adhere to slides. Cells were permeabilized with 0.1% Triton X-100 in PBS for 10 min followed by blocking with 2% bovine serum albumin in PBS for 1 h at room temperature. LeishIF4E-3 was detected using anti-LeishIF4E-3 serum (1:50) followed by incubation with goat anti-rabbit IgG DyLight 550 (1:500) (KPL). DNA was stained with DAPI (Sigma). Finally, cells were washed three times with PBS, and an antibleach mounting solution (Fluoromount-G) was added prior to their covering.

Mid-log-phase cells of the LeishIF4E-3(+/−) deletion mutant, addbacks, wild-type cells, and transgenic L. mexicana cells expressing Cas9/T7, starved for purines, along with nonstarved controls were processed for immunofluorescence microscopy (see Text S1). The slides were observed using an inverted Zeiss LSM 880 Axio-Observer Z1 confocal laser scanning microscope with an Airyscan detector. Cells were visualized using a Zeiss Plan-Apochromat oil lens objective of 63× and numerical aperture of 1.4. Z-stacked images were acquired with a digital zoom of 8× (1.8× for broad fields) using Zen Lite software (Carl Zeiss Microscopy). Images were processed using the Image J software package. A single representative section of the compiled z-projections produced by Image J software is presented in all the figures.

### *In vitro* macrophage infection assay.

L. mexicana LeishIF4E-3(+/−) deletion mutants and addback cells, wild-type cells, and transgenic parasites expressing Cas9/T7 polymerase were allowed to grow for 5 days to reach their stationary growth phase. Parasites were washed with DMEM and incubated with 10 μM carboxyfluorescein succinimidyl ester (CFSE) at 25°C for 10 min. The cells were then washed with DMEM, counted, and used to infect the RAW 264.7 macrophages at a ratio of 10:1 for 1 h. The macrophages were washed three times with PBS and once in DMEM to remove extracellular parasites. The infected macrophages were incubated for 24 h at 37°C in an atmosphere containing 5% CO_2_; shorter incubation periods (4 h) were applied to monitor entry into the cells. The infected macrophages were then processed for confocal microscopy as described above. A single representative section of Z-projections (maximum intensity) produced by Image J software is presented in all the figures. The infectivity values were determined by using the cell counting plugin in Image J. We first counted the number of infected cells in a total of 100 macrophages and then counted the number of internalized parasites within the infected cells, 4 h and 24 h following infection. Statistical analysis was generated using GraphPad Prism 5. We used the nonparametric Kruskal-Wallis test to determine significant differences in the infectivity and in the average number of parasites per infected macrophage.

### RNA-Seq analysis of LeishIF4E-3 coprecipitated mRNAs.

Mid-log-phase transgenic L. mexicana (100 ml) parasites expressing SBP-tagged LeishIF4E-3 were harvested, washed, lysed in 2 ml of PRS^+^ containing 100 units of RNase inhibitor and 1% Triton X-100, and further centrifuged at 20,000 × *g* for 20 min at 4°C. The supernatant was incubated with preequilibrated streptavidin-Sepharose beads (GE Healthcare) for 2 h. Following binding, beads were washed three times with PRS^+^ supplemented with 0.1% NP-40. Bound protein complexes were eluted in 1 ml of PRS^+^ supplemented with 5 mM biotin. The eluted material was subjected to RNA isolation using TRI reagent (Sigma) according to the manufacturer’s instructions.

**(i) RNA isolation.** The eluted material from LeishIF4E-3-SBP pulldown was mixed with 1 ml of TRI reagent (Sigma), and 0.1 ml of chloroform was added followed by vigorous mixing. The resulting mixture was centrifuged at 12,000 × *g* for 15 min at 4°C, thereby separating the mixture into three phases. The upper aqueous phase containing RNA was isolated, and 0.5 ml 2-propanol was mixed. The resulting mixture was further centrifuged at 12,000 × *g* for 10 min at 4°C to obtain an RNA pellet. The RNA pellet was washed once with 1 ml of 75% ethanol and centrifuged at 7,500 × *g* for 5 min at 4°C. The purified RNA pellet was briefly air dried, resuspended in RNase-free water, and stored at −80°C. In parallel, total cellular RNA was extracted from LeishIF4E-3-SBP-expressing cells using the abovementioned approach.

**(ii) Library preparation and RNA sequencing.** RNA that was extracted from pulled-down LeishIF4E-3 and the total RNA were subjected to the TruSeq RNA sample preparation protocol (Illumina), according to the manufacturer’s instructions. All the RNA samples were subjected to quality assessment using an Agilent TapeStation 2200, followed by fragmentation to generate blunt-ended RNAs. Blunt-ended size-selected fragments of RNA were ligated to adaptors and reverse transcribed to generate the cDNA libraries. The total RNA sample was subjected to oligo(dT) enrichment to deplete rRNAs. The libraries were sequenced at the Technion Genome Center in Haifa, Israel (http://tgc.net.technion.ac.il/), using Illumina single-end sequencing technology on a HiSeq 2500 sequencer (Illumina).

**(iii) Bioinformatics, mapping, and data processing.** Single-end Illumina sequencing reads for three repeats of LeishIF4E-3 pulled-down RNA and three repeats of the total RNA were mapped to the full genome of L. mexicana MHOM/GT/2001/U1103 (40) using Bowtie 2 (41). Each of the libraries contained 56 to 72 million 51-nucleotide reads. The quality control analysis for the reads using FastQC (42) showed very high quality of the sequences. Illumina adaptors and lower-quality sequences were removed using Trimmomatic default parameters (43). Each read was matched no more than once to the genome to allow for cumulative analysis. The aligned reads were matched to annotated genes and counted using featureCounts (44). Differential expression analysis was then done using the DESeq2 R package (45). This program compares the LeishIF4E-3 pulldown libraries with the total RNA libraries by normalizing the counts for size and dispersion factors. Normalized data were fitted using a negative binomial distribution. The Wald test was used to measure significance of the coefficients. The Wald test *P* values were adjusted for multiple testing using the Benjamini-Hochberg correction (45). Genes with adjusted *P* values of <0.05 were extracted for further analysis. To account for higher certainty, we selected genes with increased expression of over 3-fold. DESeq2 analysis was then repeated using the edgeR package (46, 47), which gave matching results.

**(iv) Gene Ontology (GO) term enrichment analysis.** Enriched transcripts were subjected to Gene Ontology (GO) enrichment analysis using TriTrypDB, based on their biological processes. The threshold for GO terms was set at a 3-fold increase with a *P* value of <0.01. This treatment excluded all the general GO terms that represented the parental terms. Some of the GO terms that were included in other functional terms were shown by a single representative GO term.

### Statistical analysis.

Statistical analysis was performed using GraphPad Prism version 5. Each experiment was performed independently at least three times, and the individual values are presented as dots in the figures. For experiments with a higher number of repeats, results are expressed as mean ± SD. Statistical significance was determined using the Wilcoxon paired *t* test for matched pairs test or the Kruskal-Wallis test with Dunn’s multiple-comparison test for comparing three or more groups. Significant *P* values are marked with asterisks in the figures: *, *P* < 0.05; **, *P < *0.01; ***, *P < *0.001.

## References

[1] Sacks DL, Perkins PV 1984 Identification of an infective state of *Leishmania* promastigotes. Science 223:1417–1420. doi:10.1126/science.6701528. 6701528

[2] Rogers ME, Ilg T, Nikolaev AV, Ferguson MA, Bates PA 2004 Transmission of cutaneous leishmaniasis by sand flies is enhanced by regurgitation of fPPG. Nature 430:463. doi:10.1038/nature02675. 15269771 PMC2835460

[3] Sunter J, Gull K 2017 Shape, form, function and *Leishmania* pathogenicity: from textbook descriptions to biological understanding. Open Biol 7:170165. doi:10.1098/rsob.170165. 28903998 PMC5627057

[4] Serafim TD, Figueiredo AB, Costa PAC, Marques-da-Silva EA, Gonçalves R, de Moura SAL, Gontijo NF, da Silva SM, Michalick MSM, Meyer-Fernandes JR, de Carvalho RP, Uliana SRB, Fietto JLR, Afonso LCC 2012 *Leishmania* metacyclogenesis is promoted in the absence of purines. PLoS Negl Trop Dis 6:e1833. doi:10.1371/journal.pntd.0001833. 23050028 PMC3458635

[5] Marr J, Berens R 1985 Purine and pyrimidine metabolism in *Leishmania*. Hum Parasit Dis 1:65–70.

[6] Martin JL, Yates PA, Soysa R, Alfaro JF, Yang F, Burnum-Johnson KE, Petyuk VA, Weitz KK, Camp DG II, Smith RD, Wilmarth PA, David LL, Ramasamy G, Myler PJ, Carter NS 2014 Metabolic reprogramming during purine stress in the protozoan pathogen *Leishmania donovani*. PLoS Pathog 10:e1003938. doi:10.1371/journal.ppat.1003938. 24586154 PMC3937319

[7] Martin JL, Yates PA, Boitz JM, Koop DR, Fulwiler AL, Cassera MB, Ullman B, Carter NS 2016 A role for adenine nucleotides in the sensing mechanism to purine starvation in *Leishmania donovani*. Mol Microbiol 101:299–313. doi:10.1111/mmi.13390. 27062185

[8] Ortiz D, Valdés R, Sanchez MA, Hayenga J, Elya C, Detke S, Landfear SM 2010 Purine restriction induces pronounced translational upregulation of the NT1 adenosine/pyrimidine nucleoside transporter in *Leishmania major*. Mol Microbiol 78:108–118. doi:10.1111/j.1365-2958.2010.07328.x. 20735779 PMC2971681

[9] Clayton C 2016 Gene expression in kinetoplastids. Curr Opin Microbiol 32:46–51. doi:10.1016/j.mib.2016.04.018. 27177350

[10] Morino S, Imataka H, Svitkin YV, Pestova TV, Sonenberg N 2000 Eukaryotic translation initiation factor 4E (eIF4E) binding site and the middle one-third of eIF4GI constitute the core domain for cap-dependent translation, and the C-terminal one-third functions as a modulatory region. Mol Cell Biol 20:468–477. doi:10.1128/mcb.20.2.468-477.2000. 10611225 PMC85104

[11] Lawrence JC Jr, Abraham RT 1997 PHAS/4E-BPs as regulators of mRNA translation and cell proliferation. Trends Biochem Sci 22:345–349. doi:10.1016/S0968-0004(97)01101-8. 9301335

[12] Marr MT, D’Alessio JA, Puig O, Tjian R 2007 IRES-mediated functional coupling of transcription and translation amplifies insulin receptor feedback. Genes Dev 21:175–183. doi:10.1101/gad.1506407. 17234883 PMC1770900

[13] Yoffe Y, Zuberek J, Lerer A, Lewdorowicz M, Stepinski J, Altmann M, Darzynkiewicz E, Shapira M 2006 Binding specificities and potential roles of isoforms of eukaryotic initiation factor 4E in *Leishmania*. Eukaryot Cell 5:1969–1979. doi:10.1128/EC.00230-06. 17041189 PMC1694823

[14] Yoffe Y, Leger M, Zinoviev A, Zuberek J, Darzynkiewicz E, Wagner G, Shapira M 2009 Evolutionary changes in the *Leishmania* eIF4F complex involve variations in the eIF4E-eIF4G interactions. Nucleic Acids Res 37:3243–3253. doi:10.1093/nar/gkp190. 19321500 PMC2691829

[15] Freire ER, Dhalia R, Moura DMN, da Costa Lima TD, Lima RP, Reis CRS, Hughes K, Figueiredo RCBQ, Standart N, Carrington M, de Melo Neto OP 2011 The four trypanosomatid eIF4E homologues fall into two separate groups, with distinct features in primary sequence and biological properties. Mol Biochem Parasitol 176:25–36. doi:10.1016/j.molbiopara.2010.11.011. 21111007 PMC6736675

[16] Shrivastava R, Drory-Retwitzer M, Shapira M 2019 Nutritional stress targets LeishIF4E-3 to storage granules that contain RNA and ribosome components in *Leishmania*. PLoS Negl Trop Dis 13:e0007237. doi:10.1371/journal.pntd.0007237. 30870425 PMC6435199

[17] Ngo H, Tschudi C, Gull K, Ullu E 1998 Double-stranded RNA induces mRNA degradation in *Trypanosoma brucei*. Proc Natl Acad Sci U S A 95:14687–14692. doi:10.1073/pnas.95.25.14687. 9843950 PMC24510

[18] Lye LF, Owens K, Shi H, Murta SM, Vieira AC, Turco SJ, Tschudi C, Ullu E, Beverley SM 2010 Retention and loss of RNA interference pathways in trypanosomatid protozoans. PLoS Pathog 6:e1001161. doi:10.1371/journal.ppat.1001161. 21060810 PMC2965760

[19] Beverley SM 2003 Protozomics: trypanosomatid parasite genetics comes of age. Nat Rev Genet 4:11–19. doi:10.1038/nrg980. 12509749

[20] Zinoviev A, Manor S, Shapira M 2012 Nutritional stress affects an atypical cap-binding protein in *Leishmania*. RNA Biol 9:1450–1460. doi:10.4161/rna.22709. 23135001

[21] Goodman CA, Hornberger TA 2013 Measuring protein synthesis with SUnSET: a valid alternative to traditional techniques? Exerc Sport Sci Rev 41:107–115. doi:10.1097/JES.0b013e3182798a95. 23089927 PMC3951011

[22] Scudiero DA, Shoemaker RH, Paull KD, Monks A, Tierney S, Nofziger TH, Currens MJ, Seniff D, Boyd MR 1988 Evaluation of a soluble tetrazolium/formazan assay for cell growth and drug sensitivity in culture using human and other tumor cell lines. Cancer Res 48:4827–4833. 3409223

[23] Gossage SM, Rogers ME, Bates PA 2003 Two separate growth phases during the development of *Leishmania* in sand flies: implications for understanding the life cycle. Int J Parasitol 33:1027–1034. doi:10.1016/S0020-7519(03)00142-5. 13129524 PMC2839921

[24] Reolon LW, Vichier-Guerre S, de Matos BM, Dugué L, da Silva Assunção TR, Zanchin NIT, Pochet S, Guimarães BG 2019 Crystal structure of the Trypanosoma cruzi EIF4E5 translation factor homologue in complex with mRNA cap-4. Nucleic Acids Res 47:5973–5987. doi:10.1093/nar/gkz339. 31066441 PMC6582342

[25] Zinoviev A, Leger M, Wagner G, Shapira M 2011 A novel 4E-interacting protein in *Leishmania* is involved in stage-specific translation pathways. Nucleic Acids Res 39:8404–8415. doi:10.1093/nar/gkr555. 21764780 PMC3201875

[26] Lueong S, Merce C, Fischer B, Hoheisel JD, Erben ED 2016 Gene expression regulatory networks in *Trypanosoma brucei*: insights into the role of the mRNA‐binding proteome. Mol Microbiol 100:457–471. doi:10.1111/mmi.13328. 26784394

[27] Beneke T, Madden R, Makin L, Valli J, Sunter J, Gluenz E 2017 A CRISPR Cas9 high-throughput genome editing toolkit for kinetoplastids. R Soc Open Sci 4:170095. doi:10.1098/rsos.170095. 28573017 PMC5451818

[28] Sunter JD, Yanase R, Wang Z, Catta-Preta CMC, Moreira-Leite F, Myskova J, Pruzinova K, Volf P, Mottram JC, Gull K 2019 *Leishmania* flagellum attachment zone is critical for flagellar pocket shape, development in the sand fly, and pathogenicity in the host. Proc Natl Acad Sci U S A 116:6351–6360. doi:10.1073/pnas.1812462116. 30850532 PMC6442623

[29] Landfear SM, Tran KD, Sanchez MA 2015 Flagellar membrane proteins in kinetoplastid parasites. IUBMB Life 67:668–676. doi:10.1002/iub.1411. 26599841 PMC4662044

[30] Saada EA, Kabututu ZP, Lopez M, Shimogawa MM, Langousis G, Oberholzer M, Riestra A, Jonsson ZO, Wohlschlegel JA, Hill KL 2014 Insect stage-specific receptor adenylate cyclases are localized to distinct subdomains of the *Trypanosoma brucei* flagellar membrane. Eukaryot Cell 13:1064–1076. doi:10.1128/EC.00019-14. 24879126 PMC4135804

[31] Varga V, Moreira-Leite F, Portman N, Gull K 2017 Protein diversity in discrete structures at the distal tip of the trypanosome flagellum. Proc Natl Acad Sci U S A 114:E6546–E6555. doi:10.1073/pnas.1703553114. 28724725 PMC5559017

[32] Courret N, Fréhel C, Gouhier N, Pouchelet M, Prina E, Roux P, Antoine J-C 2002 Biogenesis of *Leishmania*-harbouring parasitophorous vacuoles following phagocytosis of the metacyclic promastigote or amastigote stages of the parasites. J Cell Sci 115:2303–2316. 12006615 10.1242/jcs.115.11.2303

[33] Forestier C-L, Machu C, Loussert C, Pescher P, Späth GF 2011 Imaging host cell-*Leishmania* interaction dynamics implicates parasite motility, lysosome recruitment, and host cell wounding in the infection process. Cell Host Microbe 9:319–330. doi:10.1016/j.chom.2011.03.011. 21501831

[34] Rittig MG, Schröppel K, Seack KH, Sander U, N’Diaye EN, Maridonneau-Parini I, Solbach W, Bogdan C 1998 Coiling phagocytosis of trypanosomatids and fungal cells. Infect Immun 66:4331–4339. 9712785 10.1128/iai.66.9.4331-4339.1998 PMC108523

[35] Vaughan S, Kohl L, Ngai I, Wheeler RJ, Gull K 2008 A repetitive protein essential for the flagellum attachment zone filament structure and function in *Trypanosoma brucei*. Protist 159:127–136. doi:10.1016/j.protis.2007.08.005. 17945531

[36] Laban A, Wirth DF 1989 Transfection of *Leishmania enriettii* and expression of chloramphenicol acetyltransferase gene. Proc Natl Acad Sci U S A 86:9119–9123. doi:10.1073/pnas.86.23.9119. 2594753 PMC298445

[37] Peng D, Tarleton R 2015 EuPaGDT: a web tool tailored to design CRISPR guide RNAs for eukaryotic pathogens. Microb Genom 1:e000033. doi:10.1099/mgen.0.000033. 28348817 PMC5320623

[38] David M, Gabdank I, Ben-David M, Zilka A, Orr I, Barash D, Shapira M 2010 Preferential translation of Hsp83 in Leishmania requires a thermosensitive polypyrimidine-rich element in the 3′ UTR and involves scanning of the 5′ UTR. RNA 16:364–374. doi:10.1261/rna.1874710. 20040590 PMC2811665

[39] Zilka A, Garlapati S, Dahan E, Yaolsky V, Shapira M 2001 Developmental regulation of heat shock protein 83 in *Leishmania* 3′ processing and mRNA stability control transcript abundance and translation is directed by a determinant in the 3′-untranslated region. J Biol Chem 276:47922–47929. doi:10.1074/jbc.M108271200. 11598129

[40] Rogers MB, Hilley JD, Dickens NJ, Wilkes J, Bates PA, Depledge DP, Harris D, Her Y, Herzyk P, Imamura H, Otto TD, Sanders M, Seeger K, Dujardin J-C, Berriman M, Smith DF, Hertz-Fowler C, Mottram JC 2011 Chromosome and gene copy number variation allow major structural change between species and strains of *Leishmania*. Genome Res 21:2129–2142. doi:10.1101/gr.122945.111. 22038252 PMC3227102

[41] Langmead B, Salzberg SL 2012 Fast gapped-read alignment with Bowtie 2. Nat Methods 9:357. doi:10.1038/nmeth.1923. 22388286 PMC3322381

[42] Andrews S 2010 FastQC: a quality control tool for high throughput sequence data. http://www.bioinformatics.babraham.ac.uk/projects/fastqc.

[43] Bolger AM, Lohse M, Usadel B 2014 Trimmomatic: a flexible trimmer for Illumina sequence data. Bioinformatics 30:2114–2120. doi:10.1093/bioinformatics/btu170. 24695404 PMC4103590

[44] Liao Y, Smyth GK, Shi W 2014 featureCounts: an efficient general purpose program for assigning sequence reads to genomic features. Bioinformatics 30:923–930. doi:10.1093/bioinformatics/btt656. 24227677

[45] Love MI, Huber W, Anders S 2014 Moderated estimation of fold change and dispersion for RNA-seq data with DESeq2. Genome Biol 15:550. doi:10.1186/s13059-014-0550-8. 25516281 PMC4302049

[46] Robinson MD, McCarthy DJ, Smyth GK 2010 edgeR: a Bioconductor package for differential expression analysis of digital gene expression data. Bioinformatics 26:139–140. doi:10.1093/bioinformatics/btp616. 19910308 PMC2796818

[47] McCarthy DJ, Chen Y, Smyth GK 2012 Differential expression analysis of multifactor RNA-Seq experiments with respect to biological variation. Nucleic Acids Res 40:4288–4297. doi:10.1093/nar/gks042. 22287627 PMC3378882

