# Supplementary figures and images for: Deletion of a Single LeishIF4E-3 Allele by the CRISPR-Cas9 System Alters Cell Morphology and Infectivity of Leishmania
Source: mSphere. 2019 Sep 4;4(5):e00450-19. doi: 10.1128/mSphere.00450-19 (PMC6731530; doi:10.1128/mSphere.00450-19)

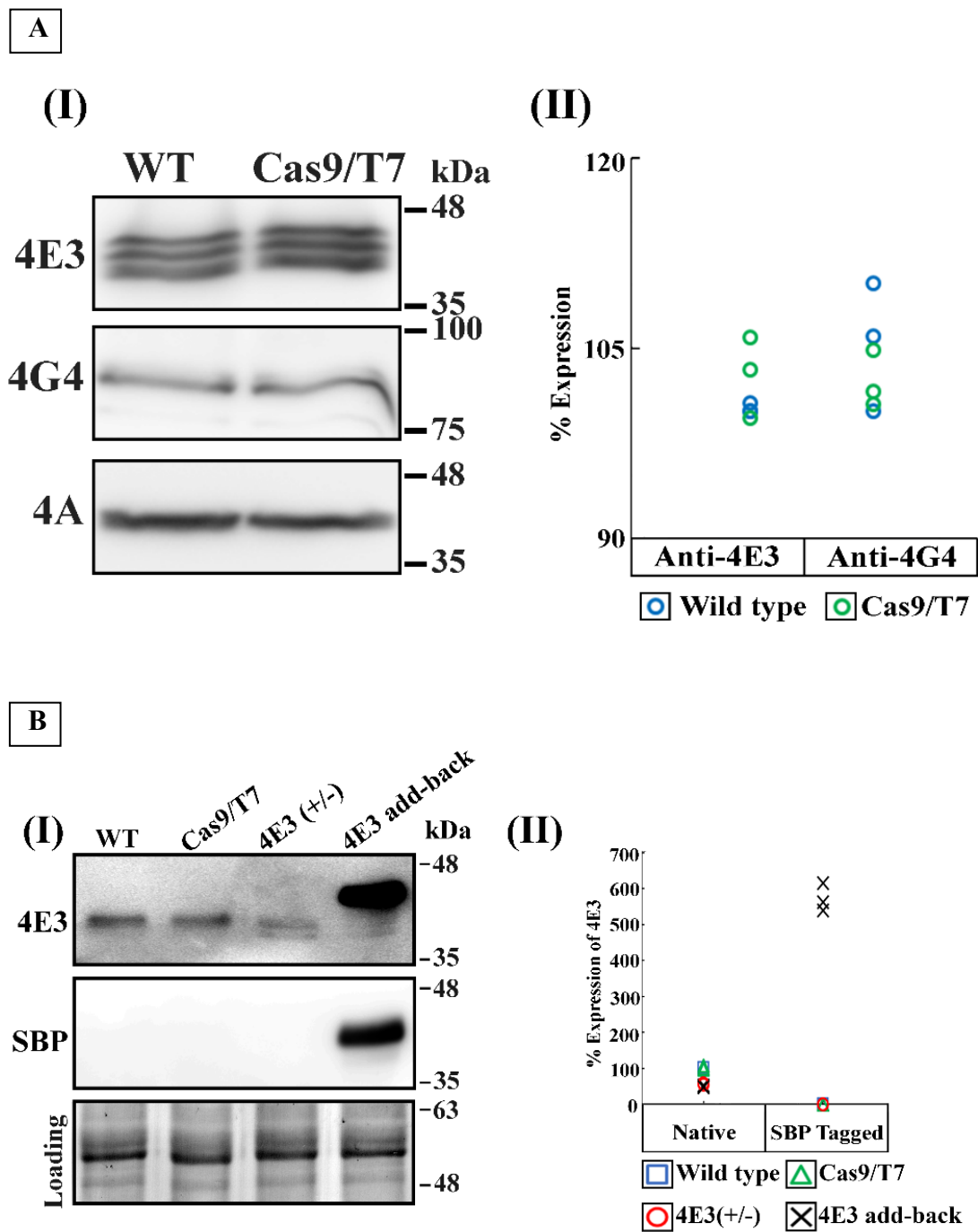

**Figure S1**

Supplement: FIG S1 [file mSphere.00450-19-sf001.pdf]

**A**

## Purine Starvation

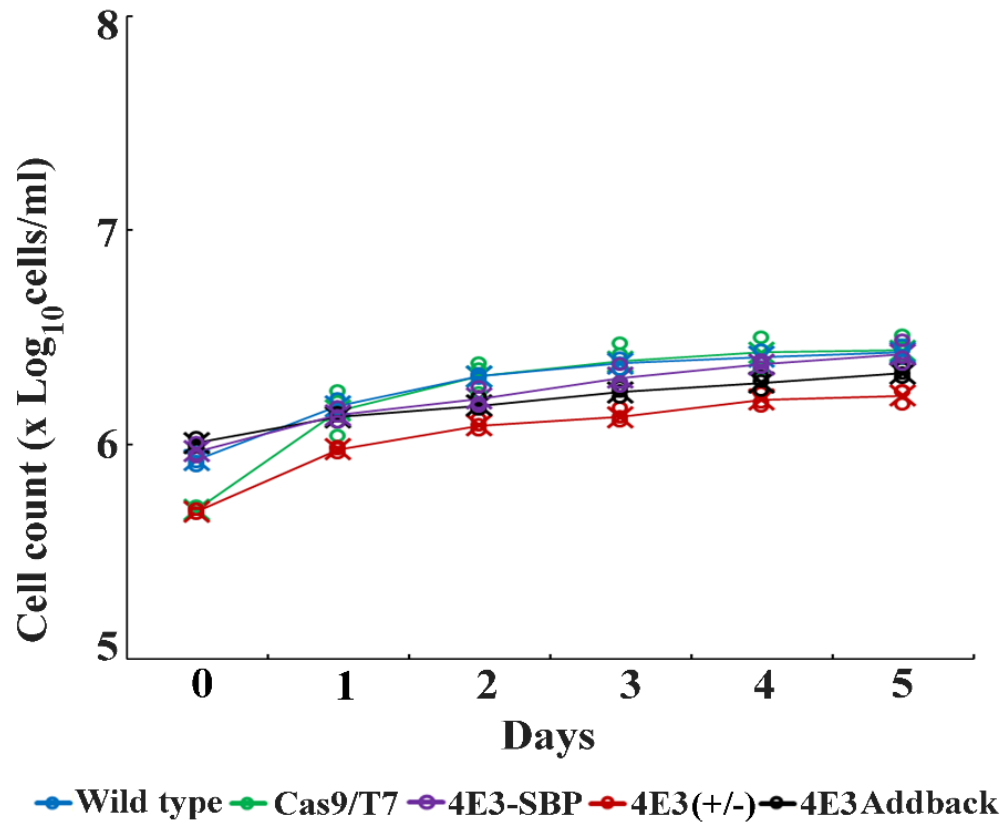

**B**

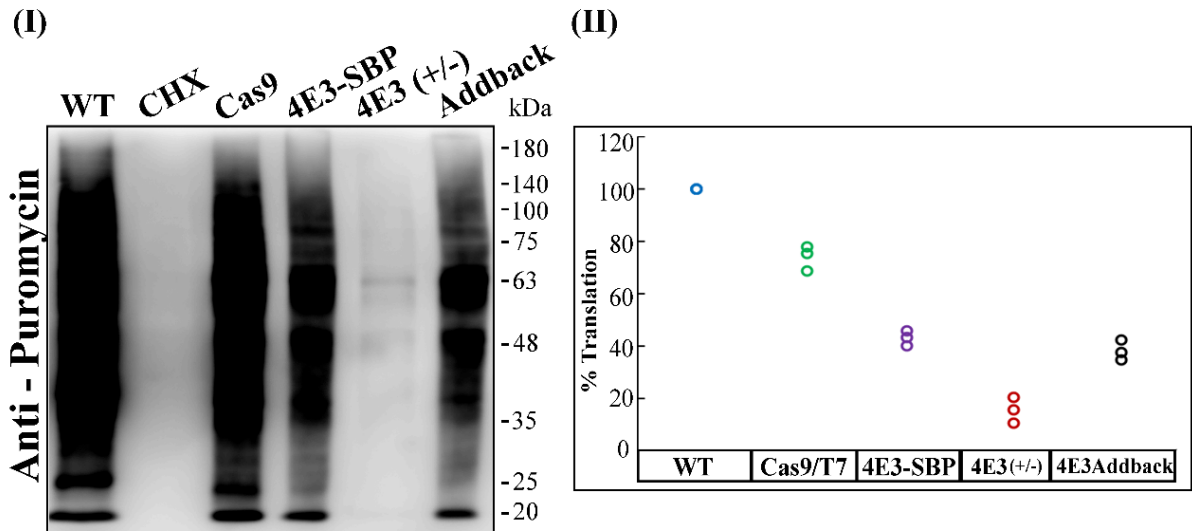

C

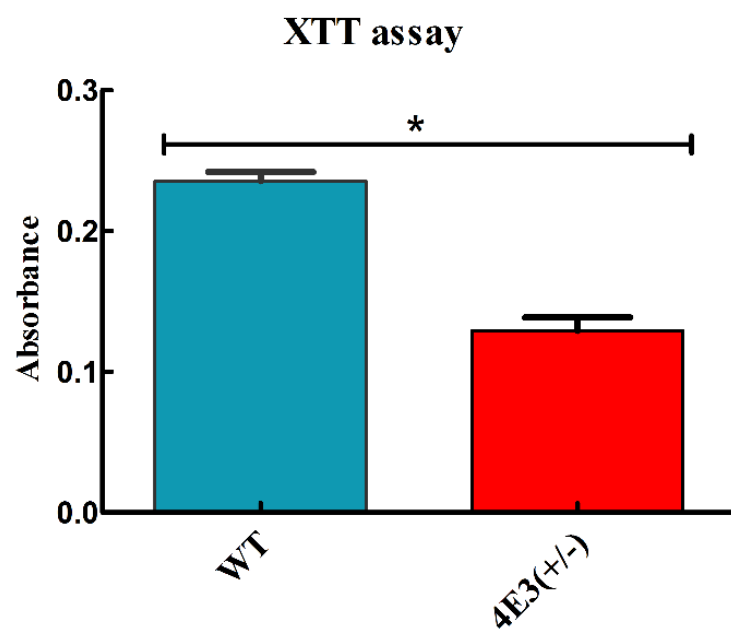

**Figure S2**

Supplement: FIG S2 [file mSphere.00450-19-sf002.pdf]

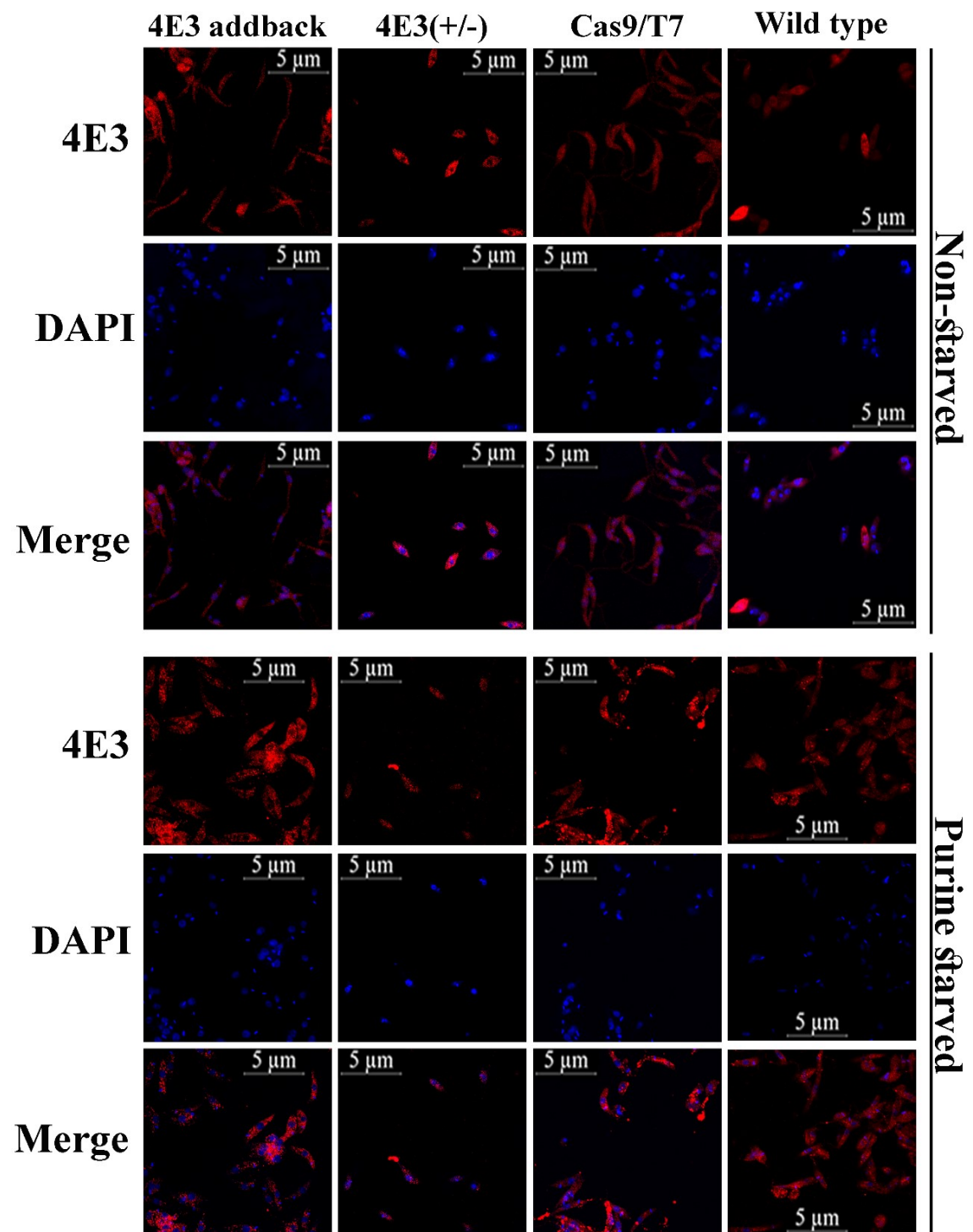

**Figure S3**

Supplement: FIG S3 [file mSphere.00450-19-sf003.pdf]

**A**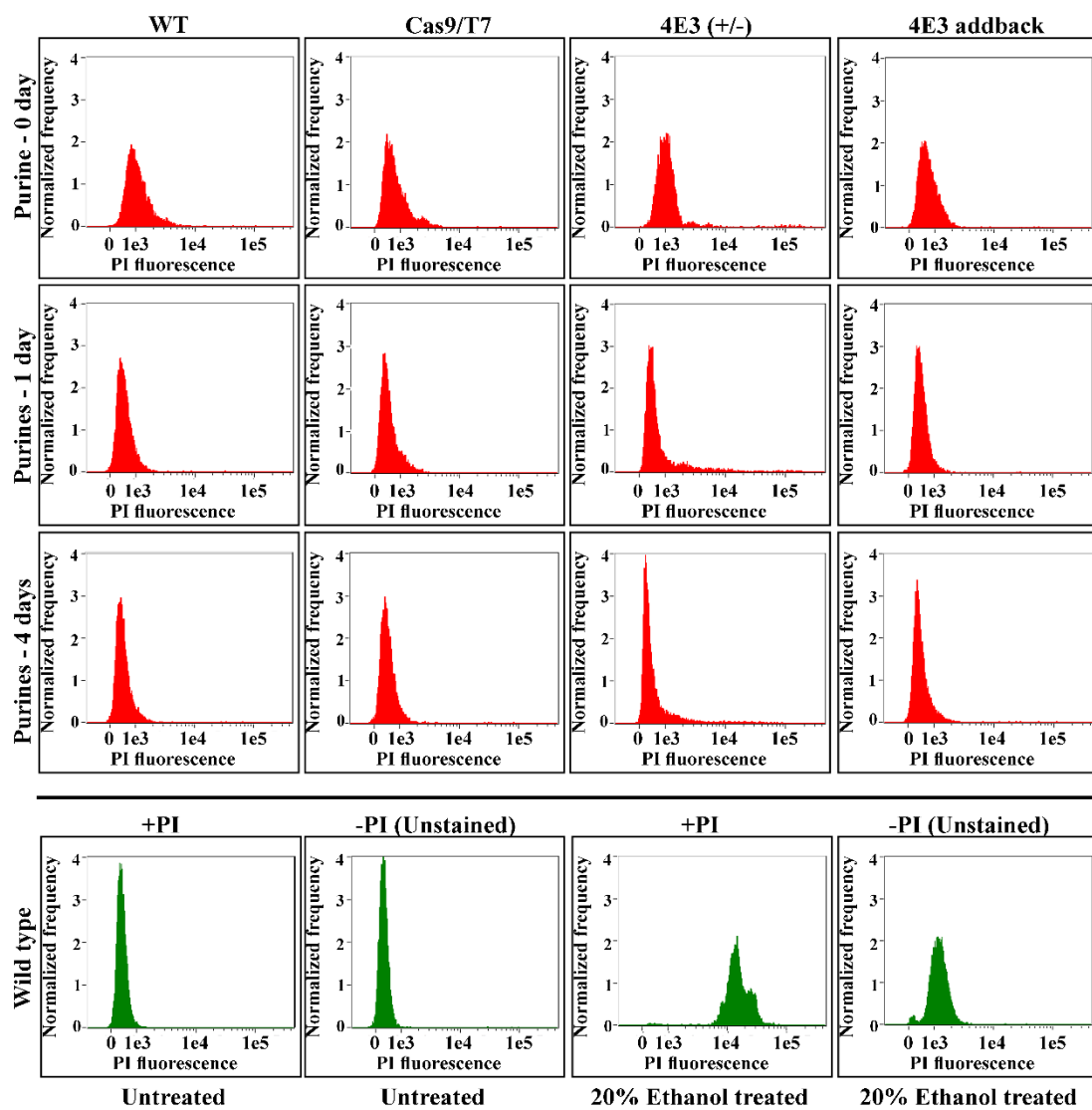

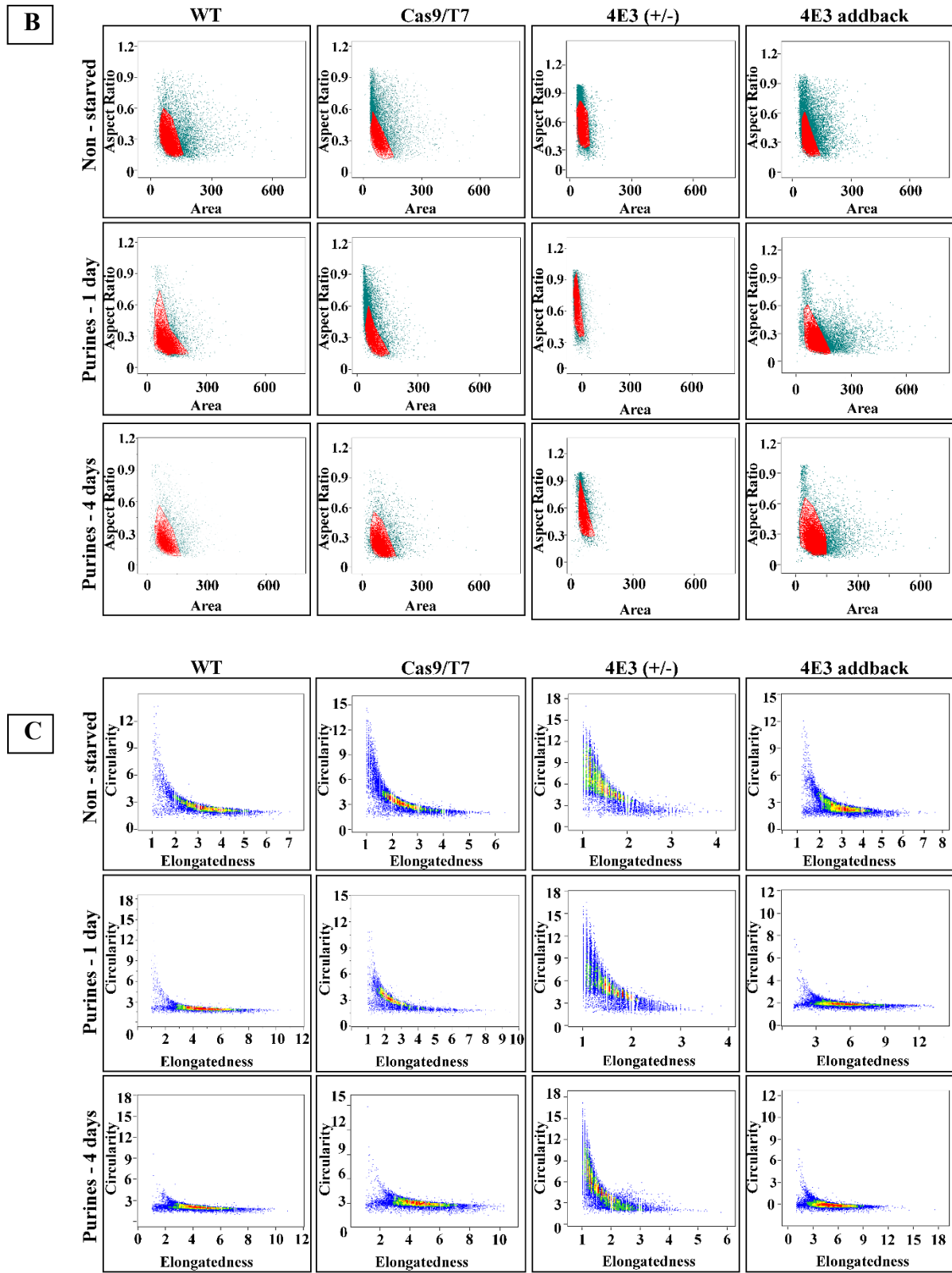

**Figure S4**

Supplement: FIG S4 [file mSphere.00450-19-sf004.pdf]

**A**

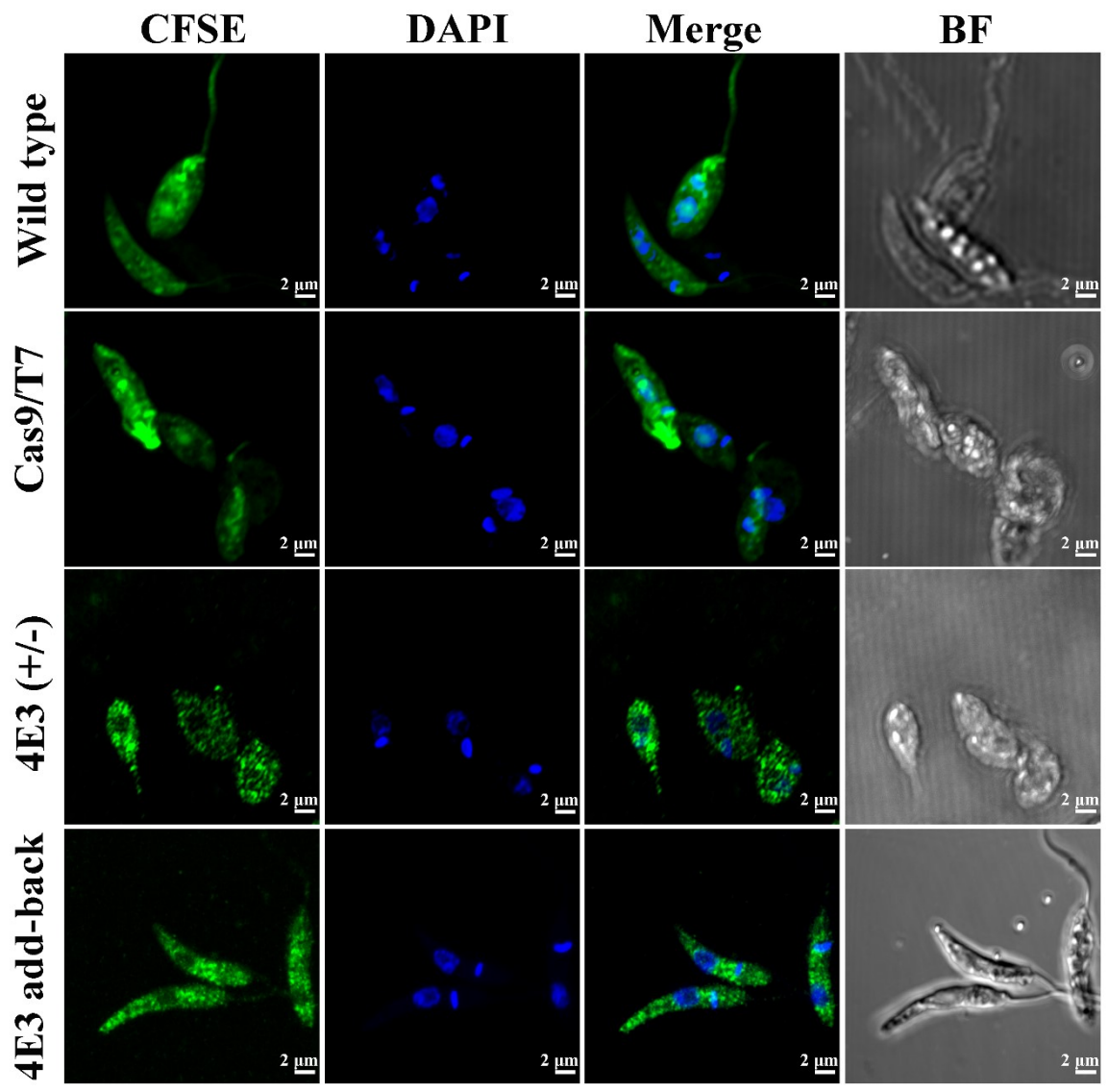

**B**

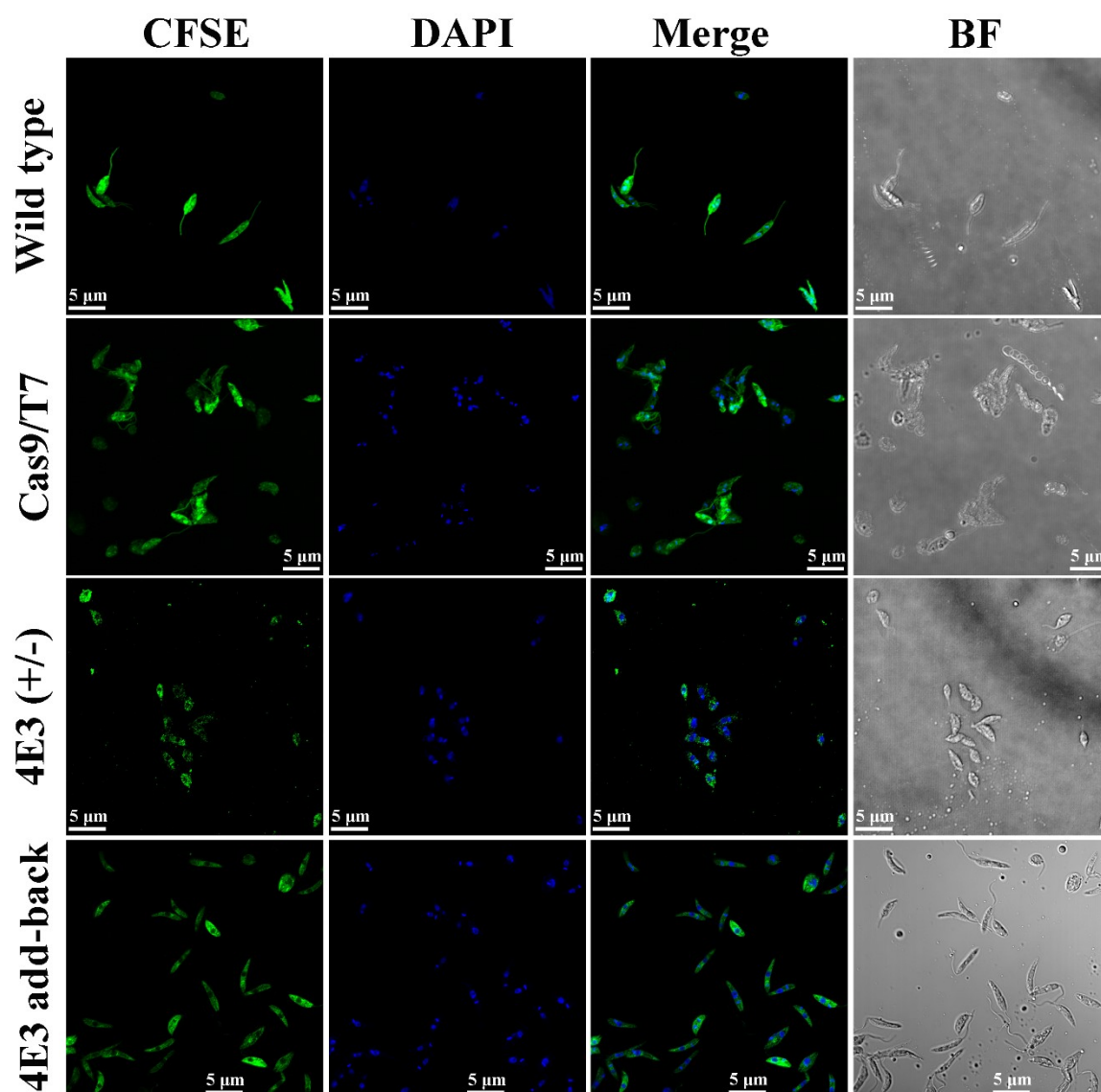

C

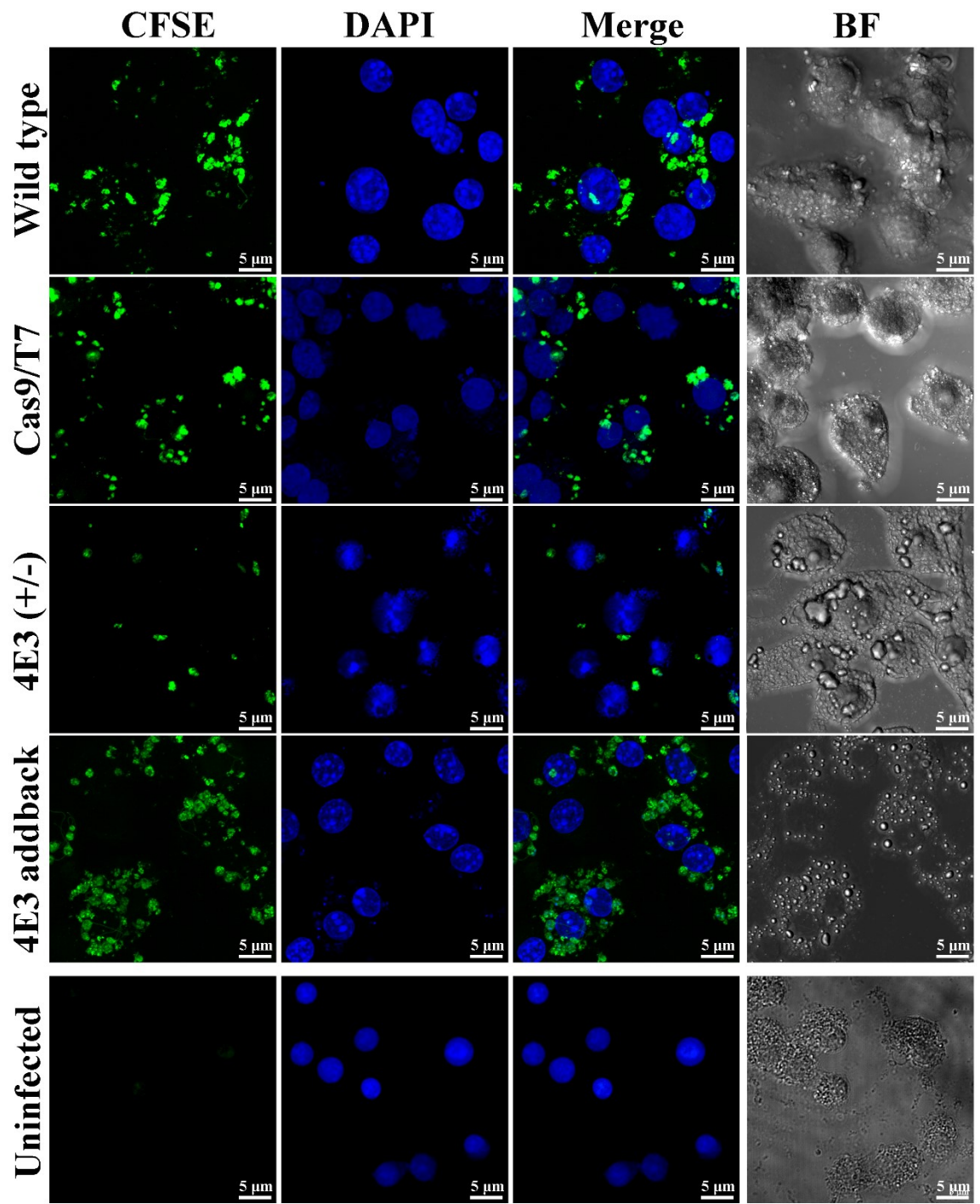

**D**

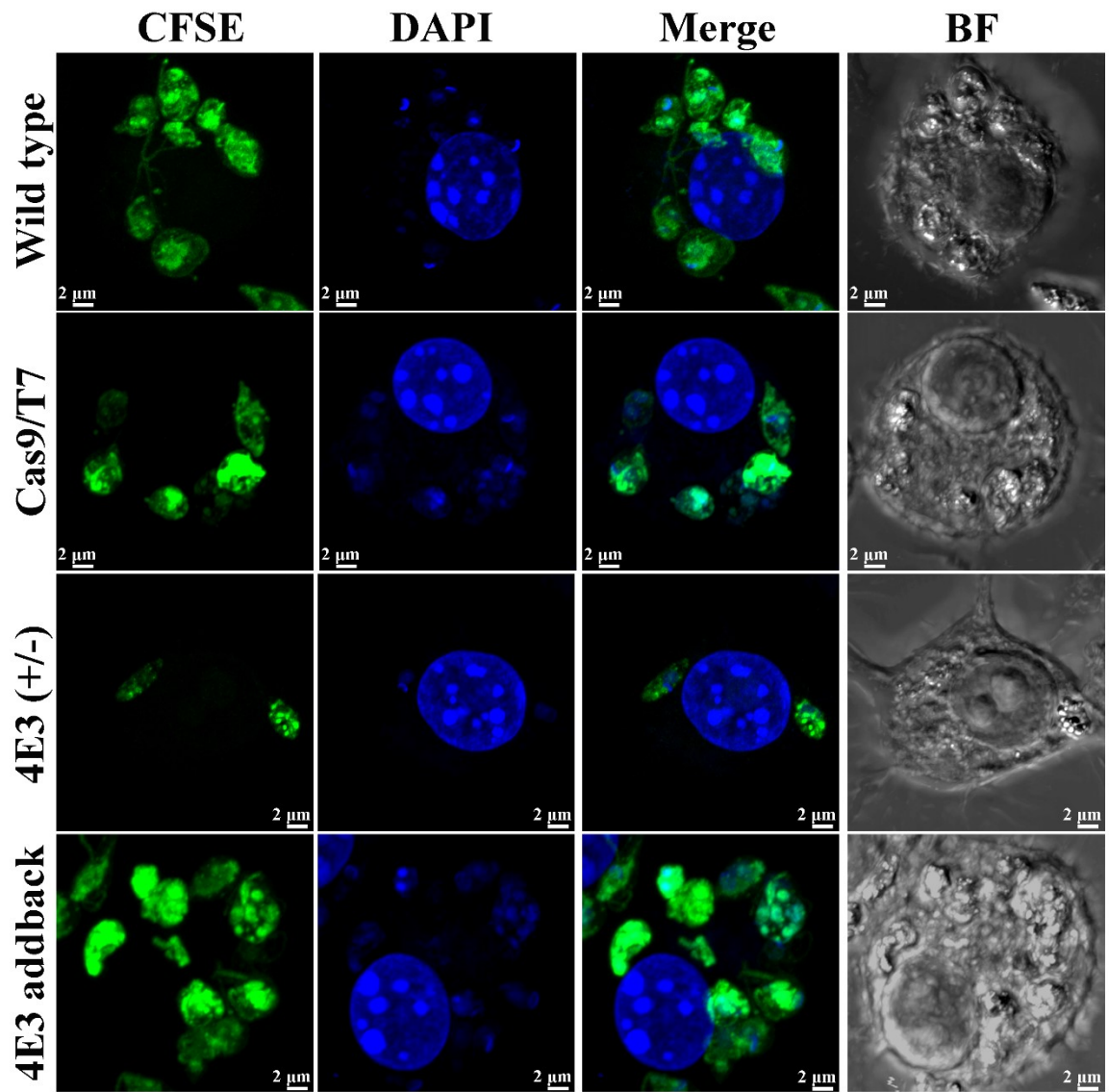

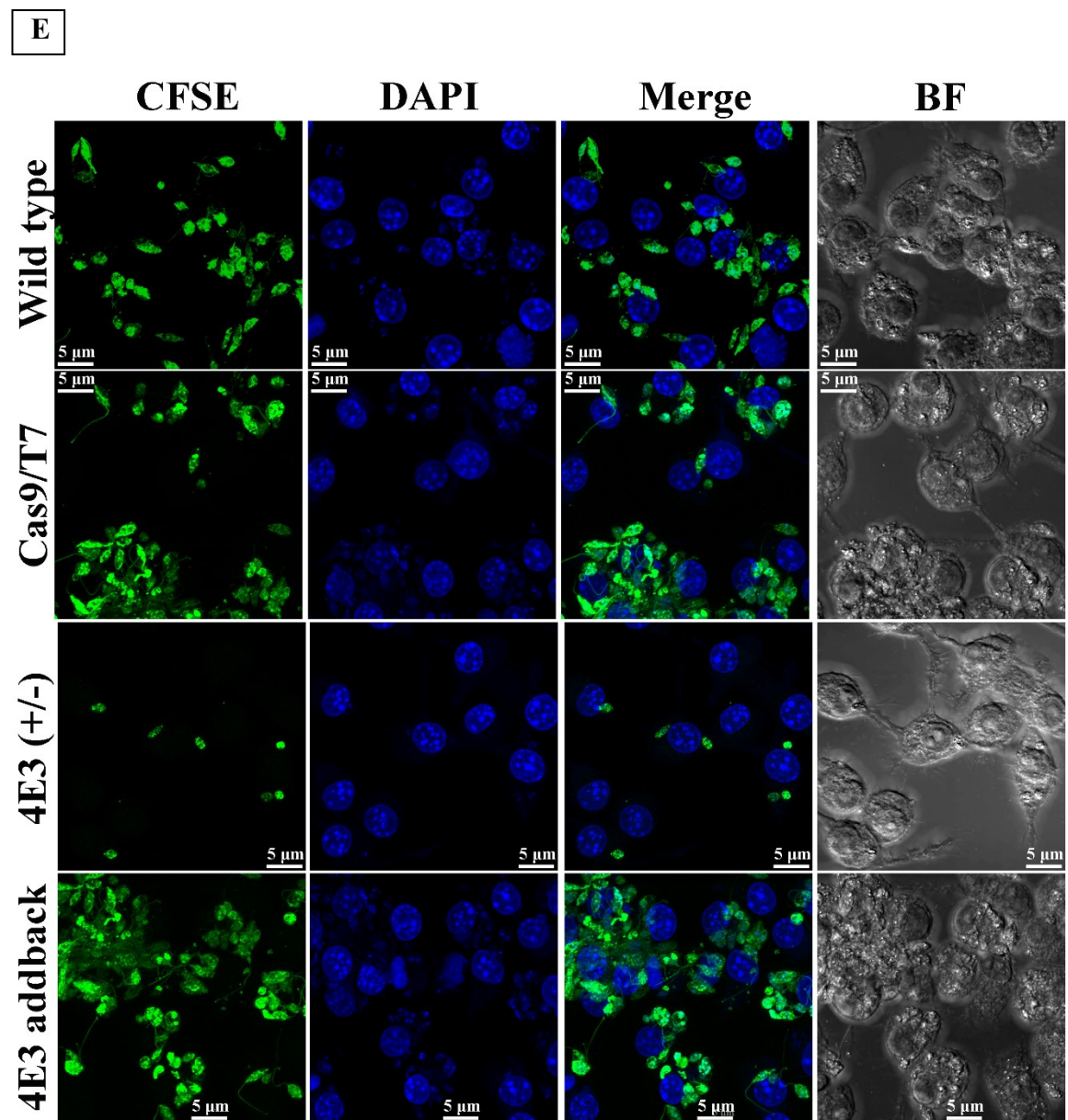

**Figure S5**

Supplement: FIG S5 [file mSphere.00450-19-sf005.pdf]
