# Supplementary material for: Deletion of a Single LeishIF4E-3 Allele by the CRISPR-Cas9 System Alters Cell Morphology and Infectivity of Leishmania
Source: mSphere. 2019 Sep 4;4(5):e00450-19. doi: 10.1128/mSphere.00450-19 (PMC6731530; doi:10.1128/mSphere.00450-19)
Supplement: TEXT S1 [file mSphere.00450-19-s0001.docx]

**Supplemental Methods**

**Cell culture**

*L. mexicana* cells were cultured in M199 medium or in Dulbecco’s modified eagle medium (DMEM), supplemented with 10% heat inactivated Foetal Calf Serum (FCS, Biological Industries), 4 mM L-glutamine, 0.1 mM adenine, 5 µg/ml Hemin, 40 mM Hepes pH 7.4, 100 U/ml penicillin and 100 µg/ml streptomycin. Non-starved control cells were grown in complete DMEM, fully supplemented as described above. Starvation for purines was performed by incubation in DMEM, except that supplements excluded adenine and dialyzed FCS was used.

RAW 264.7 macrophage cells were grown at 37^o^C in the presence of 5% CO_2_, in DMEM supplemented with 10% FCS, 4 mM L-glutamine, 0.1 mM adenine, 40 mM Hepes pH 7.4, 100 U/ml penicillin and 100 µg/ml streptomycin.

**CRISPR-Cas9 mediated knock out**

The sgRNA primers used to knock out the LeishIF4E-3 gene were originally designed using [EuPaGDT CRISPR gRNA Design Tool](http://grna.ctegd.uga.edu/) and obtained from LeishGEdit.net [1]. The sgRNA primers contained the highest scoring 20nt guide RNA sequence within 105bp upstream or downstream of the target gene. In addition, we blasted the sgRNA sequences to *L. mexicana* genome in TriTrypDB and found that the sgRNAs were highly specific for LeishIF4E-3 (E value = 0.001 and 8e^-5^). Further, we blasted the drug resistance repair cassette that contained the homology sequence to the UTR of the LeishIF4E-3, enabling recombination. The repair cassette showed an E value of 5e^-9^, suggesting very high specificity of the system.

*PCR amplification of sgRNA templates:* DNA fragments encoding LeishIF4E-3 specific 5’ and 3’ guide RNAs for cleavage upstream and downstream to the LeishIF4E-3 target gene were generated. The template for this PCR reaction consisted of two fragments, one contained the common sgRNA scaffold fragment (5’ -AAAAGCACCGACTCGGTGCCACTTTTTCAAGTTGATAACGGACTAGCCTTATTTTAACTTGCTATTTCTAGCTCTAAAAC - 3’) and the other contained the T7 RNA polymerase promoter (small letters) fused to the LeishIF4E-3 UTR sequences (capital letters) and a short sequence overlapping with the scaffold fragment (small letters). The two individual template fragments for targeting a double strand break at the 5' end of LeishIF4E-3 was (5’ –gaaattaatacgactcactataggTTCCCTCTGTGCCTAAACGCgttttagagctagaaatagc – 3’) and the template fragment targeting a double strand break at the 3' end was (5’ –gaaattaatacgactcactataggAAGGAGGCGCGAACGACATAgttttagagctagaaatagc – 3’). Each of these two fragments was annealed to the partially overlapping scaffold fragment and further amplified with two small primers derived from the T7 promoter (forward, 5’ – TTAATACGACTCACTATAGG – 3’) and the common scaffold fragment (reverse, 5’ – GCACCGACTCGGTGCCACTT – 3’). All PCR products were gel purified from agarose gels using and heated at 94ºC for 5 minutes before transfection.

*PCR amplification of the LeishIF4E-3 selection fragment:* A DNA fragment designed to repair the double-strand breaks surrounding the LeishIF4E-3 target gene was amplified by PCR. The LeishIF4E-3 specific primers were derived from the 5' and 3' endogenous UTR sequences upstream and downstream to the LeishIF4E-3 gene and the sequences from the antibiotic repair cassette, based on the LeishGEdit database (<http://www.leishgedit.net/Home.html>). The primers were (5’ – CCAGTCACACGTGTGACCCCCCTTCCACCAgtataatgcagacctgctgc – 3’ (forward) and 5’ – CTTCTCGCGATCCTTCTTCCCTCGTCTCCCccaatttgagagacctgtgc – 3’ (reverse). Capital and small letters represent the UTR sequences and the antibiotic resistance gene, respectively. The PCR reaction was performed using the pT Neo/Bla plasmid as template, generating a fragment used for repair of the double strand breaks on both sides of the gene targeted for deletion, enabling the subsequent integration of the drug resistance marker by homologous recombination at the target site.

*Diagnostic primers for PCR of the deletion cell line*

To screen for the deletion of LeishIF4E-3 following primers were used -

LeishIF4E-3 forward (5’ - ATGAACCCGTCTGCCGCTGC - 3’)

LeishIF4E-3 reverse (5’ - ACAGAAGGTGTGATCGGGC - 3’)

LeishIF4E-3 5’ UTR (5’ - CTTTTCACCATCAAGTCTCGGC - 3’)

LeishIF4E-3 3’ UTR (5’ - CACCAC GTACTCCCCACACAC - 3’)

G418 Reverse (5’ - TGGCCAGCCACGATAGCCGC – 3’)

**Generation of LeishIF4E-3 addback parasites**

The transgenic LeishIF4E-3 (+/-) deletion mutant cells were transfected with an episomal transfection vector that was based on pT-Puro that confers resistance to puromycin, and contained the SBP-tagged LeishIF4E-3 from *L. mexicana* cloned between two intergenic regions derived from the HSP83 (H) genomic cluster. Stably transfected cells were selected for resistance to puromycin. The pT-Puro-LeishIF4E-3 plasmid was generated as follows: The open reading frame of LeishIF4E-3 from *L. mexicana* was amplified using the forward- 5'-actggatccATGAACCCGTCTGCCGCAGC-3' and reverse primers – 5'-gctctagaACAGAAGGTGTGATCG-3', with BamHI and XbaI sites introduced at the 5' ends of these primers (small letters). The BamHI/XbaI PCR product was cloned into the BamHI and XbaI sites of the pX-H-SBP-H expression cassette between two intergenic regions derived from the HSP83 genomic locus [2, 3]. The fragment containing the SBP-tagged LeishIF4E-3 open reading frame (ORF) and the two HSP83 flanking intergenic regions was extracted by a Hind III cleavage, blunted, and cloned into the blunted SfoI site of pT-Puro [4]. The resulting pT-H-Puro-LeishIF4E-3-SBP-H expression vector was transfected into the heterologous LeishIF4E-3 (+/-) deletion mutant, and cells were selected for their resistance to Puromycin (50µg/ml).

**Western analysis**

Cells were harvested, washed with phosphate buffered saline [(PBS) pH 7.4] and once in post-ribosomal soup (PRS) buffer (35 mM Hepes pH 7.5, 100 mM KCl, 10 mM MgCl_2_, 1 mM DTT). The cell pellet was resuspended in PRS buffer that was supplemented with a 2x cocktail of protease inhibitors (Sigma) and 4 mM iodoacetamide (Sigma), along with the phosphatase inhibitors 25 mM sodium fluoride, 55 mM β-glycerophosphate and 5 mM sodium orthovanadate) (PRS^+^). Cells were lysed in Laemmli sample buffer and heated at 95°C for 5 minutes. Cell extracts were resolved on 12% SDS-Polyacrylamide (SDS-PAGE) gels and probed using specific antibodies against LeishIF4E-3 and its binding partner LeishIF4G-4 along with antibodies against the Streptavidin binding protein (SBP) tag. Equal protein loads were verified using specific antibodies against LeishIF4A1. All the primary antibodies were used at 1:5,000 dilutions.

**Translation assay**

Parasites were treated with 1 µg/ml of puromycin (Sigma) for 20 mins, washed twice with ice cold 1x PBS, once with PRS buffer and finally resuspended in PRS^+^ buffer. The cell pellets were lysed in Laemmli sample buffer. A control of cycloheximide treated wild type parasites demonstrate a complete translation arrest. Cell extracts (equal protein loads) were resolved on 12% SDS-PAGE. Western analysis was performed using anti-puromycin monoclonal antibodies (Developmental Studies Hybridoma Bank, University of Iowa, 1:1,000) with HRP-labeled anti-mouse secondary antibodies (KPL, 1:5,000).

**Flow cytometry analysis of *Leishmania***

*Data analysis:* IDEAS software was used to generate the quantitative measurements of the images recorded for the examined cell population. First, the focus quality of each cell was determined by measuring the gradient root mean square (RMS) value. The cells representing high value in the gradient RMS histogram were gated to select cells in focus. In the second step, single cell populations were gated from the scatter plot of aspect ratio/area to exclude cell aggregates. Further, the intensity of PI staining was used to exclude dead cells. The remaining living, single cells in focus were subjected to image analysis to determine cell morphology. To obtain cell shape, a customized adaptive erode mask was used on the bright field channel, with a coefficient of 78. We further customized this mask to exclude the flagellum from the cell shape analysis. Further, circularity and elongatedness features were measured. A pre-determined threshold value of 4 was set to define circularity. Elongatedness values represent the ratio between the cell length and width. Representative scatter plots are presented for focused single cells, and for circularity. Cell viability was measured by recording the emission of PI in the gated population. All data shown are from a minimum of three biological replicates.

**Confocal microscopy of *Leishmania* promastigotes**

*Slide preparation*: The cells were washed with PBS, fixed in 2% paraformaldehyde for 30 min, washed once with PBS and allowed to adhere to slides. Cells were permeabilized with 0.1% Triton X-100 in PBS for 10 min followed by blocking with 2% bovine serum albumin in PBS for 1 h at room temperature. LeishIF4E-3 was detected using anti-LeishIF4E-3 serum (1:50) followed by incubation with goat anti-rabbit IgG DyLight 550 (1:500) (KPL). DNA was stained used DAPI (Sigma). Finally, cells were washed three times with PBS, and an anti-bleach mounting solution (Fluoromount-G) was added prior to their covering.

**RNA co-precipitation with LeishIF4E-3**

*RNA isolation:* The eluted material from LeishIF4E-3-SBP pull-down was mixed with 1 ml of TRI Reagent (Sigma) and 0.1 ml of chloroform was added followed by vigorous mixing. The resulting mixture was centrifuged at 12,000 x g for 15 mins at 4ºC, thereby separating the mixture into three phases. The upper aqueous phase containing RNA was isolated and 0.5 ml 2-propanol was mixed. The resulting mixture was further centrifuged at 12,000 g for 10 mins at 4ºC to obtain an RNA pellet. The RNA pellet was washed once with 1ml of 75% ethanol and centrifuged at 7,500 g for 5 mins at 4ºC. The purified RNA pellet was briefly air dried, re-suspended in RNase free water and stored at -80ºC. In parallel, total cellular RNA was extracted from LeishIF4E-3-SBP expressing cells using the above-mentioned approach.

*Library Preparation and RNA sequencing:* RNA that was extracted from pulled down LeishIF4E-3 and the total RNA were subjected to the TruSeq RNA sample preparation protocol (Illumina), according to manufacturer’s instructions. All the RNA samples were subjected to quality assessment using Agilent TapeStation 2200, followed by fragmentation to generate blunt ended RNAs. Blunt ended size-selected fragments of RNA were ligated to adaptors, and reverse transcribed to generate the cDNA libraries. The total RNA sample was subjected to oligo (dT) enrichment to deplete ribosomal RNAs. The libraries were sequenced at the Technion Genome Center in Haifa, Israel (<http://tgc.net.technion.ac.il/>) using Illumina single-end sequencing technology on a HiSeq 2500 sequencer (Illumina).

*Bioinformatics, mapping and data processing:* Single-end Illumina sequencing reads for three repeats of LeishIF4E-3 pulled-down RNA and three repeats of the total RNA were mapped to the full genome of *L. mexicana* MHOM/GT/2001/U1103 [5] using Bowtie2 [6]. Each of the libraries contained 56 to 72 million 51-nucleotide reads. The quality control analysis for the reads using FastQC [7] showed very high quality of the sequences. Illumina adapters and lower quality sequences were removed using Trimmomatic default parameters [8]. Each read was matched no more than once to the genome to allow for cumulative analysis. The aligned reads were matched to annotated genes and counted using featureCounts [9]. Differential expression analysis was then done using the DESeq2 R package [10]. This program compares the LeishIF4E-3 pull-down libraries with the total RNA libraries by normalizing the counts for size and dispersion factors. Normalized data was fitted using a negative binomial distribution. The Wald test was used to measure significance of the coefficients. The Wald test *P* values were adjusted for multiple testing using Benjamini-Hochberg correction [10]. Genes with adjusted *P* value < 0.05 were extracted for further analysis. To account for higher certainty, we selected genes with increased expression of over x3 fold. DESeq2 analysis was then repeated using the edgeR package [11, 12] that gave matching results.

**References**

1. Peng D, Tarleton R. EuPaGDT: a web tool tailored to design CRISPR guide RNAs for eukaryotic pathogens. Microbial Genomics. 2015;1(4).

2. David M, Gabdank I, Ben-David M, Zilka A, Orr I, Barash D, et al. Preferential translation of Hsp83 in Leishmania requires a thermosensitive polypyrimidine-rich element in the 3′ UTR and involves scanning of the 5′ UTR. RNA. 2010;16(2):364-74.

3. Zilka A, Garlapati S, Dahan E, Yaolsky V, Shapira M. Developmental Regulation of Heat Shock Protein 83 in Leishmania 3′ processing and mRNA stability control transcript abundance, and translation is directed by a determinant in the 3′-untranslated region. J Biol Chem. 2001;276(51):47922-9.

4. Beneke T, Madden R, Makin L, Valli J, Sunter J, Gluenz E. A CRISPR Cas9 high-throughput genome editing toolkit for kinetoplastids. R Soc Open Sci. 2017;4(5).

5. Rogers MB, Hilley JD, Dickens NJ, Wilkes J, Bates PA, Depledge DP, et al. Chromosome and gene copy number variation allow major structural change between species and strains of *Leishmania*. Genome Res. 2011;21(12):2129-42.

6. Langmead B, Salzberg SL. Fast gapped-read alignment with Bowtie 2. Nature Methods. 2012;9(4):357.

7. Andrews S. FastQC: a quality control tool for high throughput sequence data 2010. Available from:

<http://www.bioinformatics.babraham.ac.uk/projects/fastqc>.

8. Bolger AM, Lohse M, Usadel B. Trimmomatic: a flexible trimmer for Illumina sequence data. Bioinformatics. 2014;30(15):2114-20.

9. Liao Y, Smyth GK, Shi W. featureCounts: an efficient general purpose program for assigning sequence reads to genomic features. Bioinformatics. 2013;30(7):923-30.

10. Love MI, Huber W, Anders S. Moderated estimation of fold change and dispersion for RNA-seq data with DESeq2. Genome biology. 2014;15(12):550.

11. Robinson MD, McCarthy DJ, Smyth GK. edgeR: a Bioconductor package for differential expression analysis of digital gene expression data. Bioinformatics. 2010;26(1):139-40.

12. McCarthy DJ, Chen Y, Smyth GK. Differential expression analysis of multifactor RNA-Seq experiments with respect to biological variation. Nucleic Acids Res. 2012;40(10):4288-97.
